# Accumulation of differentiating intestinal stem cell progenies drives tumorigenesis

**DOI:** 10.1038/ncomms10219

**Published:** 2015-12-22

**Authors:** Zongzhao Zhai, Shu Kondo, Nati Ha, Jean-Philippe Boquete, Michael Brunner, Ryu Ueda, Bruno Lemaitre

**Affiliations:** 1 Global Health Institute, School of Life Sciences, Ecole Polytechnique Fédérale de Lausanne (EPFL), Station 19, Lausanne 1015, Switzerland; 2 Invertebrate Genetics Laboratory, Genetic Strains Research Center, National Institute of Genetics, Mishima 411-8540, Japan; 3 Biochemistry Center, University of Heidelberg, Im Neuenheimer Feld 328, Heidelberg 69120, Germany

## Abstract

Stem cell self-renewal and differentiation are coordinated to maintain tissue homeostasis and prevent cancer. Mutations causing stem cell proliferation are traditionally the focus of cancer studies. However, the contribution of the differentiating stem cell progenies in tumorigenesis is poorly characterized. Here we report that loss of the SOX transcription factor, Sox21a, blocks the differentiation programme of enteroblast (EB), the intestinal stem cell progeny in the adult *Drosophila* midgut. This results in EB accumulation and formation of tumours*. Sox21a* tumour initiation and growth involve stem cell proliferation induced by the unpaired 2 mitogen released from accumulating EBs generating a feed-forward loop. EBs found in the tumours are heterogeneous and grow towards the intestinal lumen. *Sox21a* tumours modulate their environment by secreting matrix metalloproteinase and reactive oxygen species. Enterocytes surrounding the tumours are eliminated through delamination allowing tumour progression, a process requiring JNK activation. Our data highlight the tumorigenic properties of transit differentiating cells.

## Abstract (web summary)

Intestinal homeostasis is ensured by stem cell self-renewal and differentiation while alterations of these processes can lead to cancer. In this study, using *Drosophila* genetics the authors demonstrate that the loss of the transcription factor Sox21a blocks the differentiation of the intestinal stem cell progeny, which accumulate and form aggressive tumours.

## Graphical abstract

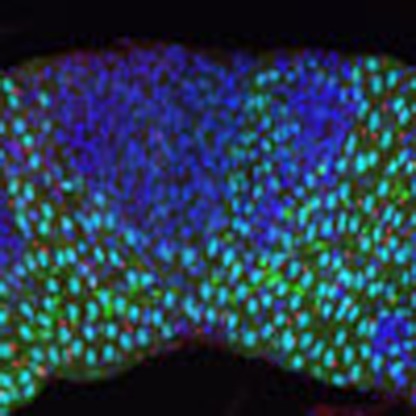

Maintenance of tissue homeostasis in the adulthood requires precise coordination of stem cell renewal and differentiation. Deregulation of these processes can lead to cancer. Stem cells live in a microenvironment and continuously receive signals from neighbouring heterologous cells composing the niche^1^. Stem cell niches are complex, heterotypic and dynamic structures^2^. Over the past few years, considerable progress has been made in elucidating how different niche factor promotes stem cell maintenance during homeostasis and contributes to tissue regeneration upon damage^3^,^4^. Stem cells usually divide asymmetrically to generate a self-renewing stem cell and a differentiating progenitor (or transit amplifying cell), which will eventually generate differentiated cells^5^. Recent studies in flies and mammals have begun to establish that these differentiating progenitors are not simply a passive intermediate between stem cell and differentiated cells, but play active roles in regulating stem cell activity and regeneration^6^,^7^,^8^,^9^,^10^.

Loss of proper differentiation is an important feature and likely a driver of cancer development^11^. Historically, mechanistic studies of human cancers and regenerative medicine have focused almost exclusively on stem cells^2^,^12^. The roles of the differentiating stem cell progeny in tumorigenesis remain largely unexplored^3^,^10^. In this study, we analyse how a defect in the differentiating program of stem cell progenies leads to tumours in the adult *Drosophila* intestine.

The adult intestine is continuously replenished by multipotent intestinal stem cells (ISCs) both in flies and mammals^4^,^12^,^13^. In the *Drosophila* midgut, ISCs differentiate into either large absorptive enterocytes or secretory enteroendocrine cells. This process involves an intermediate differentiating cell called the enteroblast (EB; Fig. 1a)^14^,^15^,^16^, analogous to the transit amplifying cell in mammalian intestines^17^. In this study, we show that *Sox21a*, a gene encoding a transcription factor of the SOX family, is required for EBs to become fully differentiated cells. Flies lacking *Sox21a* are viable but progressively develop intestinal tumours composed mainly of EBs. Using *Drosophila* genetics, we have provided a comprehensive dissection of cell–cell interactions that underlie the EB tumour initiation and progression as a result of this differentiation defect. Our data highlight a driving role of differentiating stem cell progenies in tumorigenesis. While the implication of stem cells in cancer has been the focus of intensive research, our data pinpoint the tumorigenic properties of transit differentiating cells. We speculate that the plasticity of these differentiating progenitors underlies their cancerous properties.

## Results

### *Sox21a* is necessary for EB differentiation

**Figure 1:**
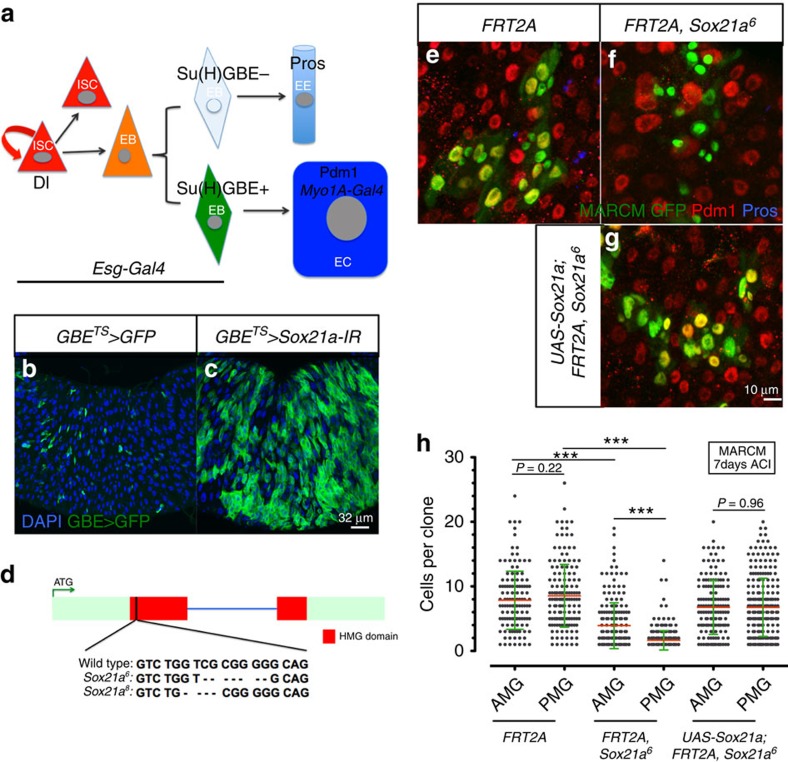
*Sox21a* is required for EB differentiation. (**a**) Model of intestinal stem cell (ISC) lineages. The markers used in this study are *Delta* (*Dl*)*-GFP/lacZ*: ISC, *escargot* (*esg*)-*Gal4* or Armadillo (Arm): progenitors (ISC+EB), *Su(H)GBE-Gal4/lacZ*: EB, Prospero (Pros): enteroendocrine cells (EE), Pdm1 or *Myo1A-Gal4*: enterocytes (EC). (**b**,**c**) Anterior midgut (AMG) of control fly and fly expressing a *Sox21a-RNAi* transgene in EBs for 14 days at 29 °C. Nuclei are stained for 4′,6-diamidino-2-phenylindole (DAPI; blue). EBs express *GBE>GFP* (green). (**d**) Schematic representation of *Sox21a* mutant alleles generated with CRISPR/Cas9 method. Sequences deleted are represented with dashed line. (**e-g**) Representative images of GFP-labelled MARCM clones from AMG of flies with indicated genotypes at 7 days after clone induction (ACI). Pdm1 (red) and Pros (blue). (**h**), Quantification of MARCM clone size for both AMG and posterior midgut (PMG) of experiments in **e**-**g**. Mean and s.e.m. are shown in **h**, with 136, 160, 221, 324, 166 and 274 clones (left to right) scored from 16 flies as a representative of three independent experiments. *P* values from Student's *t*-test (*P<0.05; **P<0.01; ***P<0.001). One representative image from 16 midguts tested in one experiment, which was repeated three times, is shown in **b**,**c** and **e-g**.

In an RNA interference (RNAi) screen for factors regulating stem cell differentiation, we identified *Sox21a*, a gene encoding a transcription factor of the SOX family with homologues implicated in stem cell regulation in mammals^18^. Silencing *Sox21a* with two independent RNAi constructs specifically in EBs using the conditional, temperature-sensitive *Su(H)GBE-Gal4 UAS-GFP tub-Gal80*^*TS*^ system (hereafter referred to as *GBE*^*TS*^)^19^ led to the accumulation of EBs in the adult (Fig. 1b,c; Supplementary Fig. 1a–c). Consistent with this, *Sox21a* is specifically enriched in the midgut of adult *Drosophila* (Supplementary Fig. 1d). Moreover, examination of the *cis*-regulatory sequences of the *Sox21a* gene also revealed an intronic enhancer that drives reporter expression in both ISCs and EBs (referred to as progenitors; Supplementary Fig. 1e–h). The expression pattern driven by this enhancer is homogenous from the anterior to the posterior midgut, and is identical to the expression of Escargot (Esg), a transcription factor with well-defined expression in progenitors^20^.

To further study the function of Sox21a, we have generated two *Sox21a* mutations using CRISPR/Cas9-mediated genome editing^21^. Both mutants carry a small deletion in the DNA-binding domain of Sox21a, the HMG domain, resulting in reading frameshift and premature stop (Fig. 1d). Thus, these alleles should be considered as null alleles. Strikingly, *Sox21a* mutant flies are viable and fertile with no apparent defects. To confirm the role of *Sox21a* in EB differentiation, we performed lineage tracing using mosaic analysis with a repressible cell marker technique (MARCM)^22^. While the wild-type clones (positively marked by green fluorescent protein (GFP)) contain both enterocytes and enteroendocrine cells, cells in *Sox21a* mutant clones along the whole midgut remained undifferentiated, as revealed by the absence of GFP-positive cells expressing the enterocyte marker Pdm1 or the enteroendocrine cell marker Prospero (Fig. 1e,f). This differentiation defect is rescued by overexpressing *Sox21a* in the mutant clones (Fig. 1g). Quantification of clone size indicated that the *Sox21a* mutation reduces ISC division with a stronger effect in the posterior compared with the anterior midgut (Fig. 1h). *Sox21a* mutant clones generated in the posterior midgut barely grew, indicating a mandatory function of Sox21a for ISC division in the posterior midgut. In contrast, the existence of large *Sox21a* mutant clones in the anterior midgut indicates that Sox21a promotes to some extent stem cell division in this region but is less essential. The presence of a wild-type copy of *Sox21a* in the mutant clones also restored normal ISC division (Fig. 1h). To further confirm the differential impact of *Sox21a* on ISC division in the anterior and posterior regions, we compared ISC proliferation rate in wild-type and *Sox21a* mutant flies overexpressing the JAK/STAT ligand unpaired 2 (Upd2) in the enterocytes with *Myo1A-Gal4 UAS-GFP tub-Gal80*^*TS*^ system (hereafter referred to as *Myo1A*^*TS*^)^23^. Unpaired are secreted proteins that have been shown to be potent inducers of ISC proliferation by activating JAK/SAT signalling in ISCs^23^,^24^. Overexpressing Upd2 strongly increased the number of mitotic ISCs in both the anterior and the posterior midgut in wild-type flies as revealed by the phospho-Histone H3 (PH3, a mitotic marker) count. In contrast, overexpressing Upd2 only increased the mitotic index in the anterior midgut of *Sox21a* mutant (Supplementary Fig. 4o,p). Collectively, our data show that *Sox21a* is essential for the differentiation of EBs into mature intestinal cells along the entire midgut. Its effect on ISC proliferation is more pronounced in the posterior midgut compared with the anterior midgut.

### Overexpression of Sox21a drives EB differentiation

**Figure 2:**
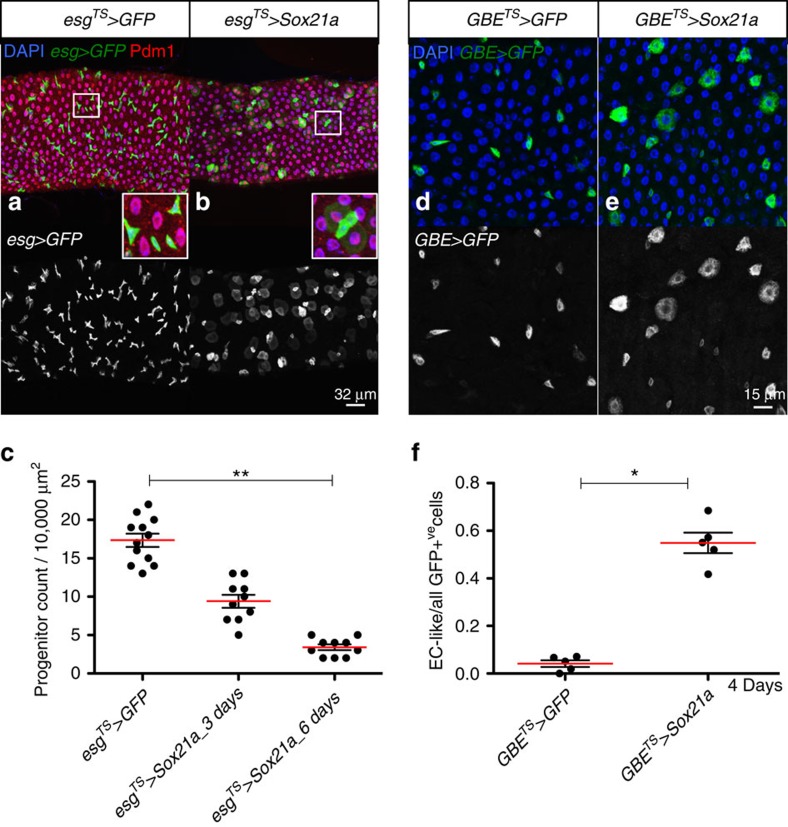
*Sox21a* is sufficient to drive differentiation. (**a**,**b**) AMG of flies overexpressing GFP (control, **a**) or *Sox21a* (**b**) in progenitor cells using *esg*^*TS*^ driver for 2 days at 29 °C. *esg>GFP* channel is also shown. Insets show a high-magnification view. Pdm1 staining is shown in red. (**c**) Quantification of the number of progenitor cells in a 10,000-μm^2^ area in AMG of control and overexpressing *Sox21a* for 3 and 6 days (*n*=12, 10 and 10, respectively). (**d**,**e**) AMG of flies overexpressing GFP (control, **d**) or *Sox21a* (**e**) in EBs using *GBE*^*TS*^ driver for 4 days at 29 °C. *GBE>GFP* channel is also shown. (**f**) Quantification of the ratio of newly formed EC-like cells to all the GFP-positive cells in AMG of control and overexpressing *Sox21a* for 4 days (*n*=5). Error bars indicate s.e.m. (**c**,**f**). *P* values from Student's *t*-test (*P<0.05; **P<0.01; ***P<0.001) are shown in **c** and **f**, and the results represent three independent experiments. One representative image from 12 midguts tested in one experiment, which was repeated three times, is shown in **a**,**b**,**d** and **e**.

Since *Sox21a* is required to generate differentiated cells, we hypothesized that overexpressing this factor might force the progenitor cells to differentiate into mature intestinal cells. To test this, we created transgenic lines that enable its overexpression via the GAL4/UAS system (Supplementary Fig. 1g). Strikingly, overexpressing *Sox21a* in the progenitor cells with *esg-Gal4 UAS-GFP tub-Gal80*^*TS*^ system (hereafter referred to as *esg*^*TS*^)^15^ was sufficient to induce their differentiation into enterocytes and cause the loss of progenitors (Fig. 2a–c). Although Sox21a is required for the differentiation of both enterocytes and enteroendocrine cells, *Sox21a* overexpression induced progenitors to differentiate into enterocytes (Pdm1 positive and polyploid), but not enteroendocrine cells (Pros positive; Supplementary Fig. 2a,b). To further delineate the role of Sox21a in progenitor differentiation, we overexpressed *Sox21a* either specifically in EBs using *GBE*^*TS*^ or in ISCs using *Dl-Gal4 UAS-GFP tub-Gal80*^*TS*^ (*Dl*^*TS*^)^19^. EBs were normally detected as small-nucleated cells with a polarized cell shape (Fig. 2d). Overexpressing *Sox21a* in EBs for 4 days transformed most of them into large polyploid and round-shaped cells, indicative of a transformation into enterocyte (Fig. 2e,f). In contrast, overexpressing *Sox21a* with *Dl*^*TS*^ in ISCs for 6 days did not induce ISC differentiation (Supplementary Fig. 2c,d). Using other insertions of the *UAS-Sox21a* transgene, we sometimes observed clusters of two to four ISCs (positive for *Dl-lacZ*) when overexpressing *Sox21a* using *esg*^*TS*^ (Supplementary Fig. 2e,f). Thus, besides inducing progenitor cells to differentiate, Sox21a may also have a role in stem cell division. We conclude that *Sox21a* is a critical factor required for the transition from EBs to mature intestinal cells in the adult midgut.

### Sox21a functions downstream of the JAK/STAT pathway

**Figure 3:**
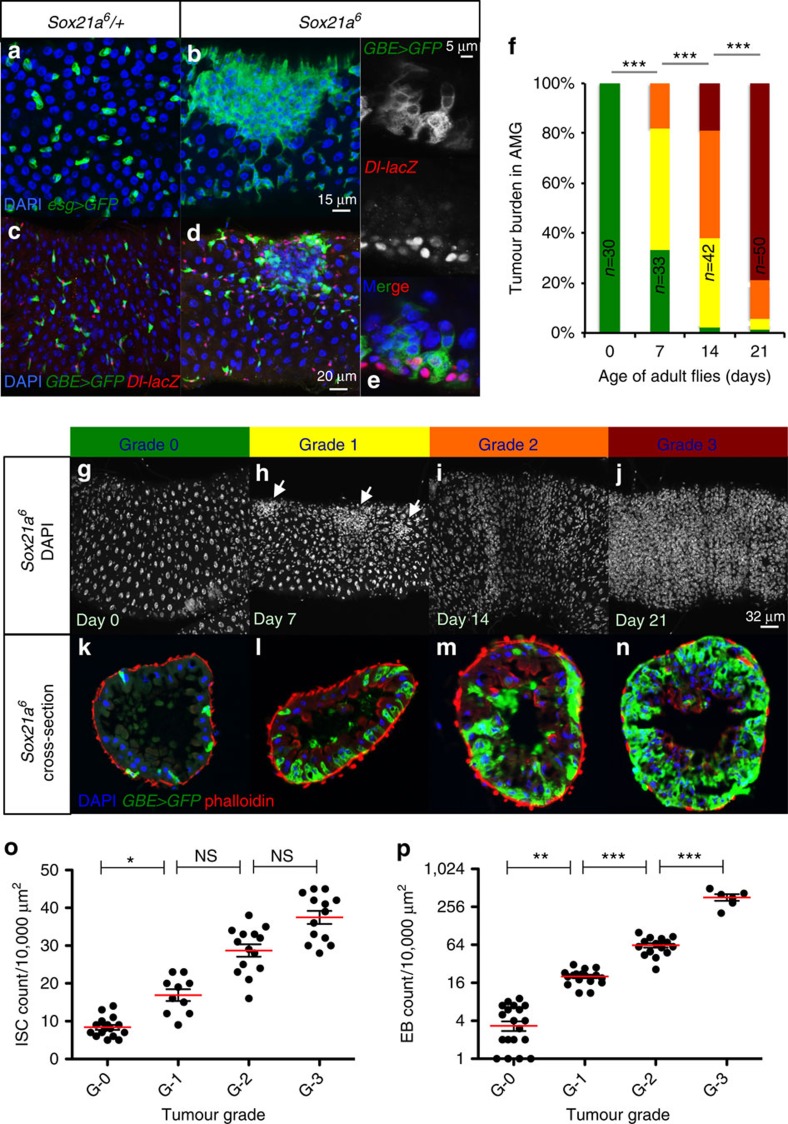
Characterization of *Sox21a* tumours. (**a**–**d**) AMG of 10-day-old *Sox21a/+* (control, **a**,**c**) and *Sox21a* flies (**b**,**d**), carrying either *esg>GFP* (**a**,**b**) or *Dl-lacZ* and *GBE>GFP* (**b**,**d**). (**e**) A confocal section to show the basal–apical (bottom-up) organization of ISCs and EBs in *Sox21a* fly. (**f**) Quantification of tumour burden of *Sox21a* flies at the indicated age at 25 °C (*n*=30, 33, 42 and 50, respectively). (**g**–**n**) Superficial view (**g**–**j**, DAPI) and cross-sections (**k**–**n**, EBs in green, phalloidin in red) of intestines from *Sox21a* flies. Tumour grade is colour-coded from green (grade 0) to red (grade 3). Arrows indicate small clusters of progenitor cells (**h**). (**o**,**p**) Quantification of the number of ISCs (**o**) and EBs (**p**) in a 10, 000-μm^2^ area from tumours of different grade (*n*=16, 10, 14 and 13, respectively in **o**; *n*=24, 17, 15 and 6, respectively in **p**). Error bars indicate s.e.m. (**o**,**p**). *P* values in **f** from *χ*^2^-test, and in **o** and **p** from Student's *t*-test (*P<0.05; **P<0.01; ***P<0.001; NS, not significant). Each plot (**f**,**o**,**p**) is representative of three biological replicates. Each image shown in **a**–**e** and **g**–**n** represents 25 flies tested in one experiment and repeated three times.

The JAK/STAT pathway plays a major role in ISC proliferation and differentiation in *Drosophila*^23^,^25^. We therefore explored the relationship between *Sox21a* and JAK/STAT in stem cell differentiation. Quantitative reverse transcription–PCR (qRT–PCR) experiments showed that *Sox21a* expression is regulated by the JAK/STAT signalling pathway. *Sox21a* expression in the midgut was lower when *Stat92E* was silenced by RNAi using the *esg*^*TS*^ driver and was higher when expressing a gain-of-function allele of JAK (*hop*^*tumL*^; Supplementary Fig. 2g). Previous study has shown that MARCM clone cells mutant for *Stat92E* were also negative for enterocyte marker Pdm1 (Supplementary Fig. 2h), consistent with a mandatory role of JAK/STAT in progenitor differentiation^23^,^25^. Moreover, overexpression of *Sox21a* restored the expression of the enterocyte marker Pdm1 in *Stat92E* null mutant clones, confirming the role of Sox21a as a major downstream effector of this pathway in mediating differentiation (Supplementary Fig. 2i). The position of Sox21a downstream of the JAK/STAT pathway and its role in EB differentiation were reinforced by two other observations. First, the expression of *esg* and its regulator *miR-8*, two genes encoding factors regulating the progenitor identity^16^,^20^, was not affected in *Sox21a* flies (Fig. 3a,b; Supplementary Fig. 3a,b). Second, the expression of Pdm1, a transcription factor specifically expressed in enterocyte^20^, was downregulated in *Sox21a* mutant EBs (see RNA sequencing (RNA-seq) experiment, described below).

### Accumulation of EBs and formation of tumour in *Sox21a* flies

A striking feature of *Sox21a* flies is the presence of large clusters of progenitors in the anterior but not the posterior midgut (Supplementary Fig. 4a–n). This regional difference is not surprising, since the *Sox21a* mutation has a differential effect on the ISC division rate in the anterior and posterior midgut. We have focused our subsequent studies on the anterior midgut to analyse the formation of these clusters. They contain both ISCs and EBs, but progressively become dominated by EBs, consistent with the function of *Sox21a* in EB differentiation (Fig. 3a–d; Supplementary Fig. 5a–h). Similar to wild-type midgut, ISCs are localized basally, while EBs are found more apically towards the lumen in *Sox21a* mutant cluster (Fig. 3e). These clusters increase in size over time and grow towards the intestinal lumen, behaving like tumours (Fig. 3g–n; Supplementary Fig. 5a–d). To quantify the tumour burden in individual midgut, we classified the tumours into four grades ranging from 0 to 3 based on their size and overgrowth (Fig. 3g–n; Supplementary Fig. 5a–d; see method part for additional information on the grading criteria). After 3 weeks at 25 °C, most *Sox21a* mutant flies contain at least one grade 3 tumour (Fig. 3f). Quantification of ISC and EB number in the tumour revealed a nearly linear increase of ISCs but an exponential increase of EBs (Fig. 3o,p; Supplementary Fig. 5a–d,e–h). This suggests that ISCs in *Sox21a* tumours are still functional and most likely divide asymmetrically to generate another self-renewing ISC and an EB blocked at this stage^26^,^27^.

### *Sox21a* tumour growth relies on ISC division

We next explored the mechanisms by which a simple defect in the differentiation program of EBs leads to tumour formation. ISCs are the only proliferating cells in the midgut in normal conditions^17^. Although a study has reported that a small portion of EBs (<5%) displays mitotic activity upon enteric *Pseudomonas entomophila* infection, EBs do not divide under basal conditions^17^. Using PH3 staining, we analysed the identity of mitotic cells in *Sox21a* mutant expressing a *GBE>GFP* to mark EBs. While we detected many ISCs undergoing mitosis, no mitotic EB was observed (*n*>100; Supplementary Fig. 6a). We next explored whether tumour growth in *Sox21a* flies is sustained by stem cell division. *Drosophila* ISC division relies on the epidermal growth factor receptor (EGFR) signalling^28^. Blocking this pathway by expressing a dominant-negative form of EGFR in progenitors of *Sox21a* flies suppressed the formation of tumour (Supplementary Fig. 6b–d). The ingestion of enteric bacteria was previously shown to stimulate ISC proliferation in *Drosophila* and promotes tumorigenesis in other models^29^. Similarly, stimulating ISC proliferation by infecting *Sox21a* flies with bacteria increased the size and the numbers of tumours (Supplementary Fig. 6e–g). These observations indicate that ISC proliferation is essential for *Sox21a* tumour formation. This dependence on stem cell division again explains why tumours are only found in the anterior midgut but not the posterior midgut where *Sox21a* is required for both EB differentiation and ISC division.

**Figure 4:**
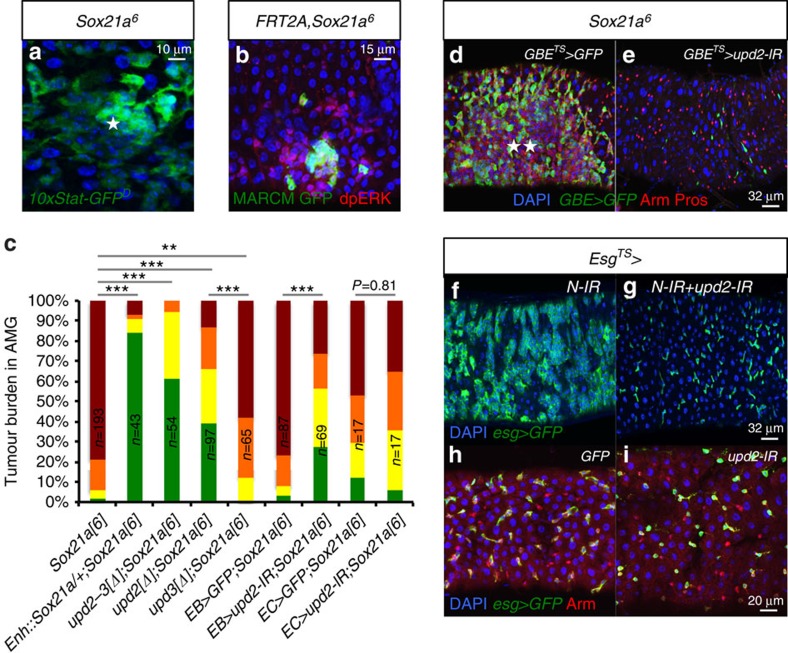
EB-derived *upd2* is essential for tumour growth and ISC maintenance. (**a**) AMG of 10-day-old *Sox21a* flies stained for *10xSTAT-GFP*^*D*^(green) shows increased JAK/STAT activity in the tumour (indicated by a star). (**b**) AMG containing GFP-labelled *Sox21a* mutant clone stained for dpERK (red) at 16 days ACI. dpERK staining is observed around the *Sox21a* clone. (**c**) Tumour burden of flies with the indicated genotypes kept at 25 °C (mutant analysis) or 29 °C (overexpression analysis) for 21 days. *Enh::Sox21a* is a rescue construct with *Sox21a* under the control of its own enhancer sequence. ‘*EB*>' refers to the EB driver *GBE*^*TS*^, and ‘*EC>*' refers to the enterocyte driver *Myo1A*^*TS*^. Numbers of flies scored for each genotype are indicated. (**d**,**e**) Expressing GFP (**d**, control) or *upd2-RNAi* (**e**) in EBs of *Sox21a* flies placed for 21 days at 29 °C. Gut was stained with Armadillo (Arm; red, plasma membrane for progenitors) and Prospero (Pros, red, nuclear, for enteroendocrine cells). EBs are marked by *GBE>GFP* (green). (**f-g**) AMG of flies depleted for *Notch* (*N*) alone (**f**) or in combination with *upd2* (**g**) for 4 days at 29 °C. The expression of *upd2-IR* in progenitors reduced tumour formation. (**h**,**i**) AMG of flies expressing GFP (control, **h**) or *upd2-RNAi* (**i**) in the progenitor cells using the *esg*^*TS*^ driver for 14 days at 29 °C shows that *upd2* is required for basal stem cell maintenance. Progenitors are shown by *esg>GFP* in green (**f**–**i**) and by Arm immunostaining (**h**,**i**). *P* values in **c** (repeated three times) from *χ*^2^-test (*P<0.05; **P<0.01; ***P<0.001). Each other individual image shown in **a**,**b** and (**d**–**i**) represents 20 flies tested in one experiment repeated three times.

Interestingly, ISC proliferation was markedly increased in the neighbourhood of *Sox21a* tumours (Supplementary Fig. 7a). Consistent with this, use of *10xStat-GFP*^*D*^ reporter gene reveals higher JAK/STAT activity in the tumour (Fig. 4a). In addition, *Sox21a* mutant cells generated via MARCM triggered Ras/MAPK signalling in neighbouring wild-type cells, as revealed by a staining of phosphorylated ERK (dpERK) (Fig. 4b). Thus, *Sox21a* mutant cells induce a local hyperplasia by stimulating division in adjacent ISCs (Supplementary Fig. 7b). This suggests that EBs composing *Sox21a* tumours send a signal to neighbouring ISCs to drive their proliferation.

### EB-derived Upd2 is essential for *Sox21a* tumour growth

ISC proliferation can be induced upon expression of secreted ligands of the EGFR pathway (Spitz, Vein and Keren), the JAK-STAT pathway (unpaired 1, 2 and 3) and the Wingless pathway (Wg)^6^,^23^,^24^,^28^,^30^,^31^. To identify the factor stimulating stem cell division in *Sox21a* tumour, we applied a candidate gene approach by knocking down genes encoding these ligands in either EBs with *GBE*^*TS*^ or in enterocytes with *Myo1A*^*TS*^. Depletion of the JAK/STAT ligand *upd2* by RNAi in EBs but not in enterocytes strongly reduced tumour formation in *Sox21a* flies (Fig. 4c–e). Similarly, *upd2; Sox21a*-double mutant flies displayed a reduction in tumour burden (Fig. 4c). In contrast, inhibiting the other factors in EBs or enterocytes (Upd1, Keren and Wg) did not impair *Sox21a* tumour formation (Supplementary Fig. 8a,b). It should be noted that *upd3* mutation and to a lesser extent depletion of *Spitz* in EBs had a modest effect on *Sox21a* tumours (Fig. 4c; Supplementary Fig. 8a). This indicates that ISC proliferation is mostly induced by Upd2 released from *Sox21a* EBs composing the tumour. The stimulation of ISC proliferation by EB-derived Upd2 produces more differentiation-defective EBs, providing a feed-forward loop underlying the expansion of *Sox21a* tumours. We hypothesize that tumours are initiated in *Sox21a* flies upon stochastic clustering of EBs, leading to local increase of the mitogen Upd2. This mechanism would explain the random localization of tumours observed in the anterior midgut of *Sox21a* flies.

In addition to *Sox21a* mutation, loss of Notch signalling in progenitors has been shown to induce tumours in the *Drosophila* midgut^14^,^15^,^32^. In contrast to the *Sox21a* EB tumours, *Notch* tumours are composed of ISCs that fail to differentiate into EBs. We have investigated the role of Upd2 in *Notch* tumour formation. Simultaneous depletion of *upd2* and *Notch* by RNAi in the progenitors also suppressed *Notch* tumours (Fig. 4f,g), further emphasizing the role of Upd2 in tumour growth. Importantly, silencing *upd2* in progenitor cells in an otherwise wild-type background led to a decrease in ISC numbers, pointing to a role of Upd2 in basal level stem cell maintenance (Fig. 4h–i).

### Mmp2 is required for *Sox21a* tumour progression

**Figure 5:**
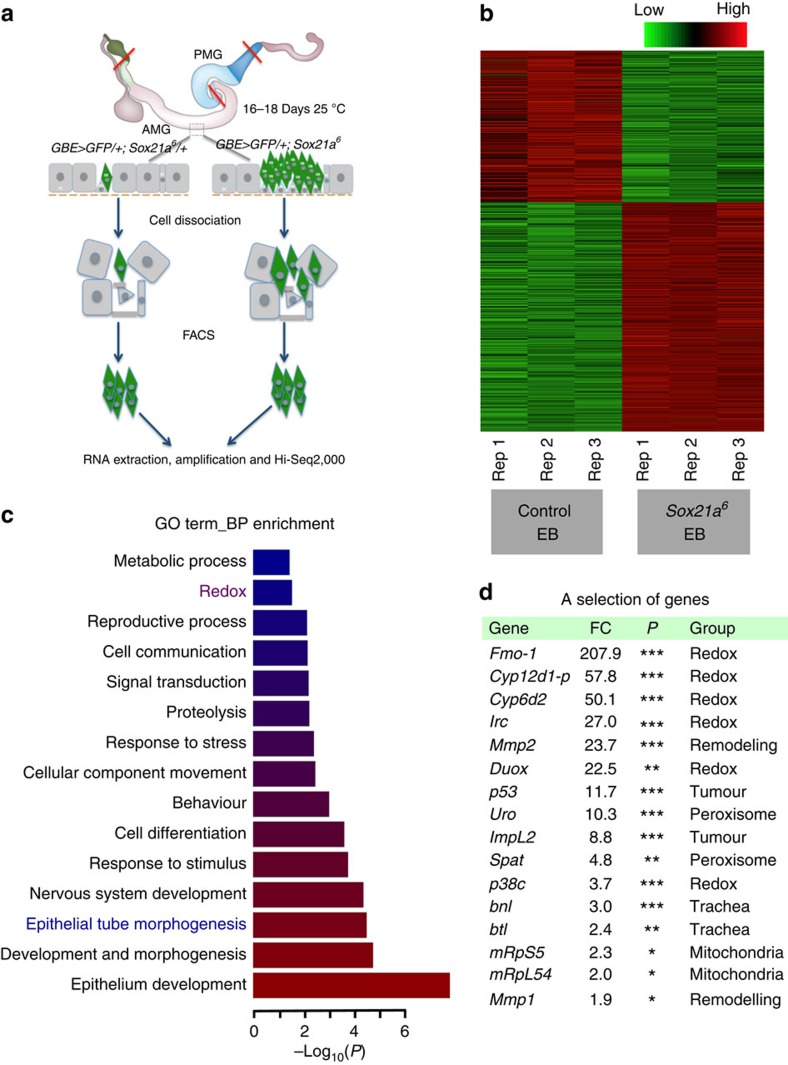
EB-specific transcriptomics. (**a**), Transcriptional profiling of *Sox21a/+* (control) and *Sox21a* EBs by RNA-seq was performed with messenger RNA isolated from FACS-sorted EBs based on the presence of *GBE>GFP* fluorescent signal (see Method part for details). (**b**) Clustering of 1,080 differentially expressed genes (*P*<0.05, Robinson and Smyth exact test) between *Sox21a/+* and *Sox21a* EBs revealed that biological repeats (triplicates) cluster together. Columns correspond to replicate and rows to different genes. Relative expression level is indicated by the colour key shown at the top. (**c**) The 1,080 genes with differential expression were classified based on their gene ontology (GO) terms for biological process (BP). Enrichment of each GO term is shown with the *P* value. Red terms are the most significantly enriched ones. Redox and epithelial tube morphogenesis, described afterwards in the paper, are in red and blue, respectively. (**d**) A selection of genes upregulated in *Sox21a* EBs. Fold change (FC), the range of *P* values (*<0.05; **<0.01; ***<0.001) and the gene function groups are shown.

We then investigated how a defect in the differentiation program transforms EBs into an aggressive tumour. For this, we compared gene expression of fluorescence-activated cell sorting (FACS)-sorted EBs of wild-type and *Sox21a* flies by RNA-seq^33^ (Fig. 5a). Of 1,080 differentially expressed genes (*P*<0.05, Robinson and Smyth Exact Test), 668 genes were reproducibly upregulated and 412 genes downregulated in *Sox21a* EBs compared with control (Fig. 5b). Gene ontology analysis of the RNA-seq data set revealed enrichment in genes involved in epithelia tube morphogenesis and redox homeostasis in *Sox21a* EBs (Fig. 5c). Many genes that were previously shown to be associated with tumorigenesis in other models were also identified in *Sox21a* tumour. For instance, the genes *ImpL2*, an insulin/insulin-like growth factor antagonist recently reported to mediate tumour-induced organ wasting^34^,^35^, and *p53*, which reprograms tumour metabolism^36^, were both upregulated in *Sox21a* EBs (Fig. 5d). Increased expression of *breathless*/fibroblast growth factor receptor (FGFR; *btl*) in *Sox21a* EBs was confirmed using *btl-Gal4 UAS-actGFP* (referred to as *btl>actGFP*; Supplementary Fig. 9a–f). While *btl>actGFP* never labels intestinal cells in wild-type midgut (Supplementary Fig. 9a), expression of the *btl>actGFP* reporter was observed in the anterior but not posterior midgut of *Sox21a* flies, in regions where tumours form (Supplementary Fig. 9b–f). The observation that some but not all the *Sox21a* EBs express *btl>actGFP* highlights the cellular heterogeneity of *Sox21a* tumours (Supplementary Fig. 9c).

**Figure 6:**
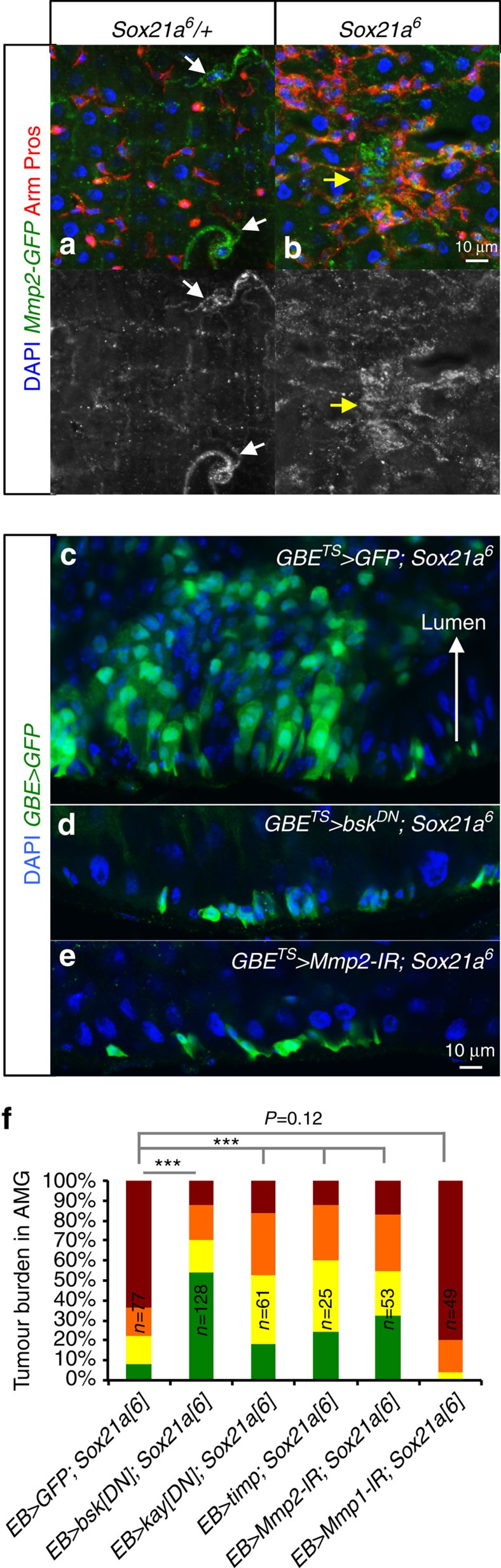
Mmp2 is required for tumour progression. (**a**,**b**), Expression of Mmp2 detected with an endogenous *Mmp2-GFP* fusion protein in the AMG of 10-day-old *Sox21a/+* (control, **a**) and *Sox21a* flies (**b**). Arm (red, plasma membrane) and Pros (red, nuclear). Note that Mmp2 is only expressed in tracheal cells in the control (arrows in a), while it is expressed in the midgut progenitor cells (Arm positive) in *Sox21a* flies (arrow in **b**). (**c**–**e**) Confocal sections of *Sox21a* intestines expressing GFP (control, **c**), *bsk*^*DN*^ (**d**) or *Mmp2-RNAi* (**e**) in EB for 21 days at 29 °C. EBs are in green. (**f**), Tumour burden in AMG of flies with the indicated genotypes monitored at 21 days at 29 °C. Kayak (Kay) encodes the c-fos component of the JNK transcription factor AP-1. *timp* encodes a protein inhibitor of matrix metalloproteinase. Numbers of flies scored for each genotype (in biological triplicates) are indicated in **f**. *P* values from *χ*^2^-test (*<0.05; **<0.01; ***<0.001). Each other individual image shown in **a**–**e** represents 20 flies tested in one experiment, which was repeated three times.

Interestingly, genes encoding two matrix metalloproteinases, matrix metalloproteinase 2 (*Mmp2*) and to a lesser extent matrix metalloproteinase 1 (*Mmp1*) were upregulated in *Sox21a* EBs (Fig. 5d). Use of an endogenous *Mmp2-GFP* fusion^37^ confirmed an increased expression of Mmp2 specifically at the tumour site (Fig. 6a,b). *Mmp1* and *Mmp2* are downstream effectors of the JNK pathway that mediate tumour invasiveness in an imaginal disc tumour model^38^,^39^,^40^. Inactivating the JNK pathway by expressing a dominant-negative form of JNK (*basket, bsk*), depleting *Mmp2* (but not *Mmp1*) or expressing the tissue inhibitor of metalloprotease (*timp*) in EBs of *Sox21a* flies reduced tumour burden and growth towards the lumen (Fig. 6c–f). Of note, Mmp2 but not Mmp1 was previously shown to be required for the invasive growth of larva air sac/trachea into tissues^41^.

### Tumour progression requires JNK activation in enterocytes

**Figure 7:**
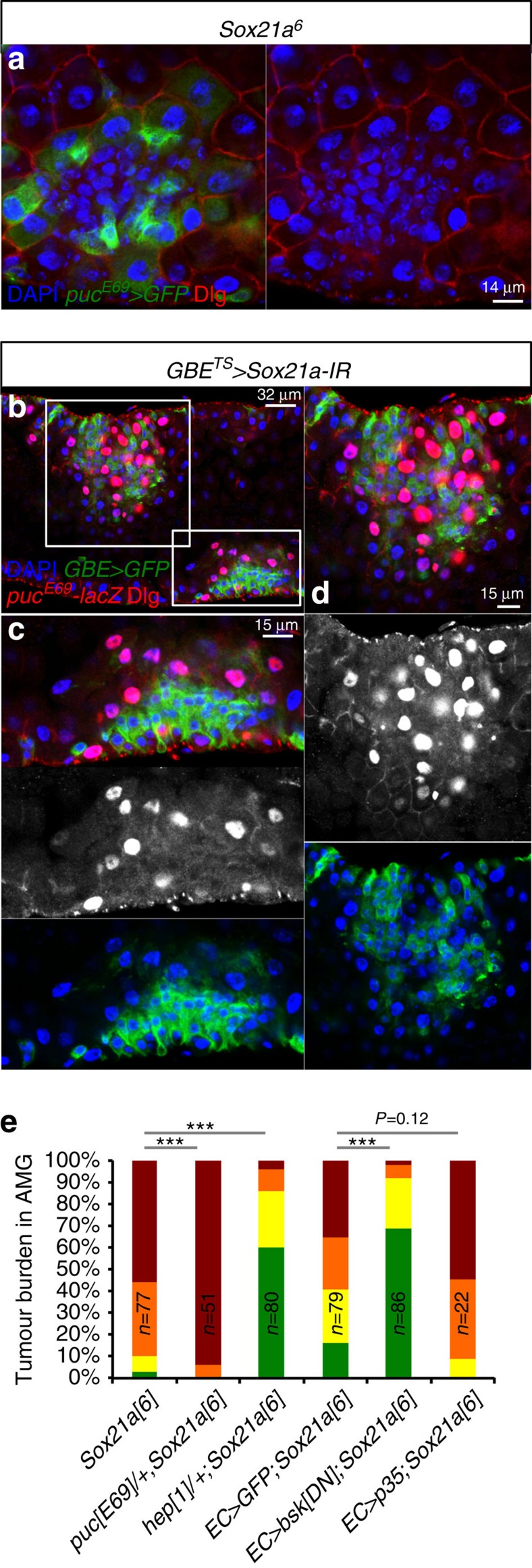
Tumour-induced elimination of surrounding enterocytes requires JNK activation. (**a**) Two-week-old *Sox21a* intestine stained with the JNK activity reporter *puc*^*E69*^*-Gal4 UAS-GFP* (in green) and the basolateral cell-polarity marker Discs large (Dlg, in red). (**b**–**d**) Confocal section of intestine with EB-specific depletion of *Sox21a* for 2 weeks, stained with the JNK activity reporter *puc*^*E69*^*-lacZ* (red, nuclear) and Dlg (red, plasma membrane). EBs (*GBE>GFP*) are in green. (**c**,**d**) High-magnification view of two tumours defined in **b**. (**e**) Tumour burden in AMG of flies with the indicated genotypes monitored at 21 days. Flies were kept at 25 °C (for mutants) or 29 °C (for misexpression). Numbers of flies scored for each genotype (in biological triplicates) are indicated in **e**. *P* values from *χ*^2^-test (*<0.05; **<0.01; ***<0.001). Each other individual image shown in **a**–**d** represents 20 flies tested in one experiment, which was repeated three times.

Tumour progression in *Sox21a* flies was associated with the elimination of neighbouring enterocytes, as shown by the progressive disappearance of 4,6-diamidino-2-phenylindole (DAPI)-stained polyploid cells (Fig. 3g–j; Supplementary Fig. 5a–d). Tumour-induced elimination of normal cells has been observed in other cases and probably reflects a common feature of aberrantly proliferating cells^42^,^43^. It is reminiscent of cell competition in *Drosophila*, a type of short-range cell–cell interaction, where the fitter cells eliminate the unfit cells by activating JNK signalling^44^. Using a *puckered* enhancer trap (*puc-lacZ* and *puc-Gal4*) as readout for JNK activity, we found that JNK signalling was induced in enterocytes surrounding *Sox21a* tumours from *Sox21a* flies or flies with EB-specific depletion of *Sox21a* by RNAi (Fig. 7a,b). Interestingly, JNK activation in enterocytes was coupled with the loss of the cell-polarity marker Discs large (Dlg) (Fig. 7a). In *Notch* ISC tumour^43^, flanking enterocytes are eliminated by delamination into the lumen. Similarly, confocal microscopy revealed the presence of delaminating enterocytes in the lumen of *Sox21a* flies at the vicinity of tumour (Fig. 7c). However, in *Sox21a* tumour, EBs were also found intercalated with enterocytes (Fig. 7d). Several observations indicate that JNK activation in enterocytes flanking *Sox21a* mutant tumours is essential for tumour progression. First, *Sox21a* flies lacking one copy of *hemipterous* (*hep*), a gene encoding the JNK kinase, have decreased tumour burden. In contrast, *puc*^*E69*^*/+* heterozygote flies with enhanced JNK activity display an increase of tumour burden of *Sox21a* flies (Fig. 7e). Second, inactivation of JNK signalling specifically in enterocytes by expressing a dominant-negative form of JNK greatly suppressed tumour formation and the presence of delaminating enterocytes (Fig. 7e). Moreover, the elimination of enterocytes nearby the tumours does not involve caspase-dependent apoptosis, as tumour progression was not affected by expressing the caspase inhibitor P35 (Fig. 7e). Collectively, our data show that *Sox21a* tumour progression involves the elimination of enterocytes by JNK activation independent of caspase activation.

### Increase of ROS activity at the vicinity of *Sox21a* tumour

**Figure 8:**
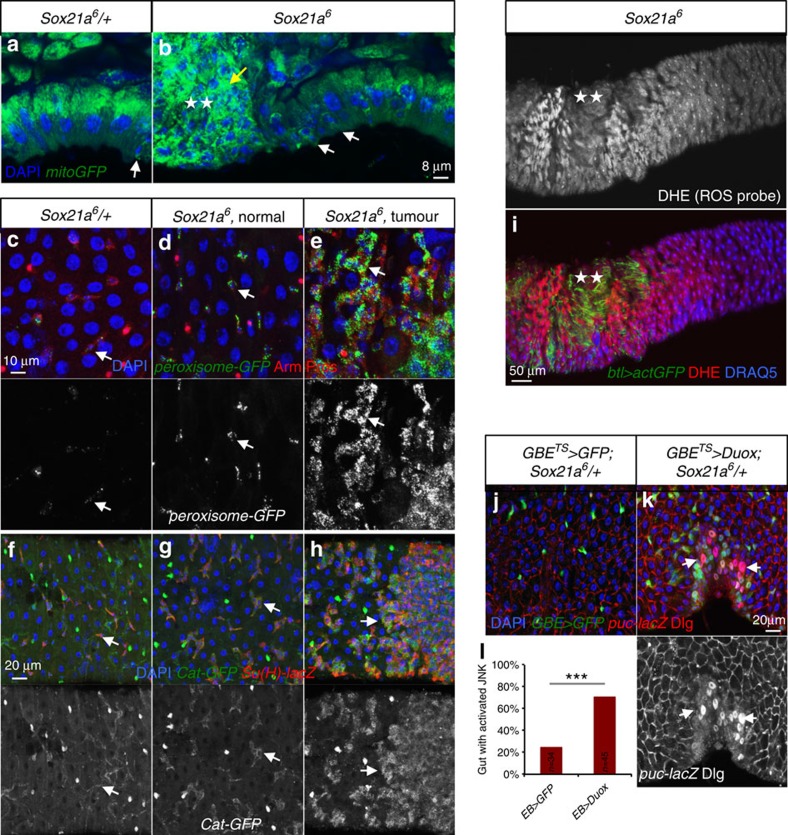
Increase of ROS at the border of *Sox21a* tumours. (**a**,**b**) Confocal sections of 2-week-old *Sox21a/+* (control, **a**) and *Sox21a* (**b**) intestine expressing the mitochondrial-targeting GFP (*mitoGFP*, green) driven by *Act5C-Gal4*. While *mitoGF*P signals are found in progenitors of wild-type and *Sox21a* flies (white arrows in **a** and **b**), a higher level of mitochondrial signal (yellow arrow in **b**) was observed at the site of *Sox21a* tumour (indicated with two stars). (**c**–**e**,**f**–**h**) Two-week-old *Sox21a/+* (control, **c**,**f**) and *Sox21a* (**d**,**e**,**g**,**h**) intestine either expressing a GFP fusion protein targeting the peroxisome (*peroxisome-GFP*, green) driven by *Act5C-Gal4* (**c**–**e**) or a Catalase (Cat)-GFP protein trap (**f**–**h**). Shown are regions from the AMG of *Sox21a* flies without (**d**,**g**) or with tumour (**e**,**h**). Signals corresponding to peroxisomes and Cat are both enriched in progenitor cells of *Sox21a* flies (*Sox21a*^*6*^, normal) compared with control flies (*Sox21a*^*6*^/+), and are further enhanced in *Sox21a* tumour sites (*Sox21a*^*6*^, tumour; indicated by arrows). Progenitors are marked by Arm (red, plasma membrane) in **c**–**e**. EBs are marked by *Su(H)-lacZ* in **f**–**h**. (**i**) ROS distribution in *Sox21a* intestine as revealed by dihydroethidium (DHE) staining of unfixed tissue. Cells are visualized by the live-cell DNA-dye DRAQ5 (blue) and tumour by the expression of *btl>actGFP* (green, indicated by two stars). (**j**–**l**) Representative images (**j**,**k**) and quantification (**l**) of *puc-lacZ* expression in *Sox21a/+* flies either expressing GFP (control, **j**,**l**) or *Duox* (**k**,**l**) in EBs for 10 days at 29 °C. Examples of enterocyte with activated JNK signalling are indicated by arrows (**k**). Numbers of flies scored for each genotype (in biological triplicates) are indicated in **l**. *P* values from *χ*^2^-test (*P<0.05; **P<0.01; ***P<0.001). Each individual image shown in **a**–**k** represents 12 flies tested in one experiment (in three biological replicates).

Reactive oxygen species (ROS) have been implicated in JNK activation and induction of cell death in *Drosophila*. For example, feeding flies with the ROS-generating compound Paraquat causes cellular damage and JNK activation in the midgut^45^,^46^. A recent study reported that ROS from apoptotic cells propagate to and activate JNK in the nearby cells^47^. This raises the possibility that tumour-derived ROS contribute to JNK activation in surrounding enterocytes, facilitating their elimination. We therefore investigated the role of ROS in tumorigenesis in *Sox21a* flies. Gene ontology analysis of the RNA-seq data set revealed enrichment in genes involved in redox homeostasis in *Sox21a* EBs (Fig. 5c). For instance, *Sox21a* EBs display increased expression of several *Cytochrome P450* genes, the NADPH oxidase *Dual oxidase* (*Duox*) and its regulator, the MAPK *p38c* (refs 48, 49), as well as several genes encoding mitochondria and peroxisome components (Fig. 5d). Mitochondria and peroxisomes are two main sources of intracellular ROS^50^. Using reporter constructs (*mitoGFP* and *peroxisome-GFP*), we observed an increase in mitochondrial and peroxisome signals at the tumour site, evocating a shift of metabolism (Fig. 8a–e; Supplementary Fig. 10a,b). Increase of peroxisomes in *Sox21a* EBs is further supported by the expression of Catalase (Cat), a protein localized to peroxisome (Fig. 8f,h). Consistent with these observations, *in vivo* ROS detection using dihydroethidium (DHE) revealed a gradient of ROS peaking at the periphery of *Sox21a* tumours (Fig. 8i). Surprisingly, the level of ROS at the tumour site was lower, suggesting that EB tumour cells were less exposed to ROS compared with neighbouring enterocytes. Reporter genes and immunostaining analysis showed that many enzymes involved in ROS detoxification, including Catalase, Glutathione *S*-transferase D1 (GstD) and Superoxide dismutase 2 (SOD2) are enriched in progenitors of *Sox21a* flies (Fig. 8g,h; Supplementary Fig. 10c–e). These observations suggest that these progenitor cells have an increased capacity to deal with ROS, and explain the lower level of ROS in *Sox21a* tumour.

We then investigated the role of ROS in tumour formation by feeding *Sox21a* flies with an antioxidant, *N*-acetylcysteine amide (AD4). We observed that *N*-acetylcysteine amide-fed *Sox21a* flies have reduced tumour burden, although the difference with untreated control did not reach statistic significance (Supplementary Fig. 8c). Interestingly, overactivation of the ROS-producing enzyme Duox specifically in EBs of wild-type flies led to increased JNK activity in the flanking cells, and often resulted in local hyperplasia (Fig. 8j–l). In these experiments, foci of JNK activation were not observed around individual EBs but only around clustered EBs (Fig. 8k). Clustering of EBs expressing *Duox* might thus lead to a local increase of ROS above a threshold sufficient to trigger JNK activity. While the relevance of ROS in *Sox21a* tumour progression requires further investigation, our data raise the possibility that tumour-derived ROS non-cell autonomously contribute to JNK activation and elimination of flanking enterocytes.

## Discussion

**Figure 9:**
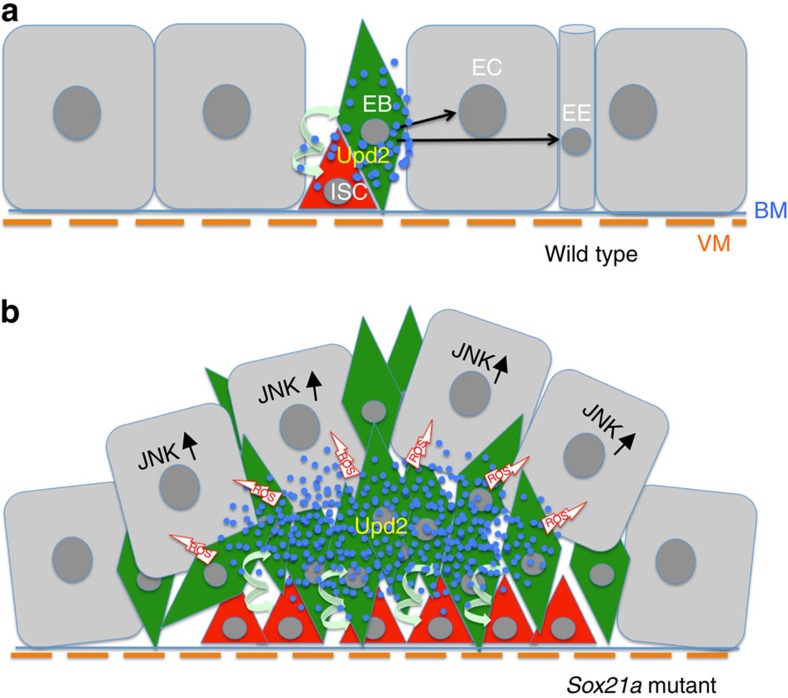
Model of *Sox21a* tumour initiation and progression. (**a**) Schematic representation of a wild-type intestinal epithelium. Intestinal stem cells (ISCs) are localized basally close to the basement membrane (BM) and visceral muscles (VMs). ISCs self-renew and differentiate to generate differentiating progenitors, the enteroblasts (EBs), which will then further differentiate into either enterocytes (ECs) or enteroendocrine (EE) cells. Progenitors express Upd2 (blue dots) stimulating basal level ISC turnover. (**b**) The *Sox21a* mutation blocks the differentiation of EBs to ECs or EE cells, resulting in the accumulation of EBs. Clustered EBs create a centre with high Upd2 level that simulates ISCs division, generating more differentiation-defective EBs. EB tumour cells eliminate flanking ECs by delamination probably under the action of ROS and mechanical pressure. Elimination of flanking ECs, a process requiring JNK signalling activation, further provides space and mitogens allowing tumour progression.

In this work, we report the functional characterization of a new regulator of ISC differentiation, introduce a novel tumour model and provide mechanistic insights on how tumour may arise from a simple defect in the differentiation program of stem cell progenies (Fig. 9a,b).

Our data show that Sox21a, a previously uncharacterized transcription factor of the SOX family, plays a major role in the terminal differentiation of ISC progenies. Although the *Drosophila* genome encodes eight Sox genes, *Sox21a* is the only one enriched in the midgut. *Sox21a* is specifically expressed in progenitor cells along the entire midgut, and acts downstream of the JAK/STAT signalling to permit the transformation of EBs into enterocytes and enteroendocrine cells. Although Sox21a is required for both differentiated cell types, overexpression of Sox21a drives differentiation into enterocytes. It cannot be excluded that high level of Sox21a due to overexpression approach favours enterocyte rather than the enteroendocrine cell fate. Overexpression of Sox21a rescues the differentiation marker Pdm1 that is lost in JAK/STAT-deficient clones, demonstrating that Sox21a contributes significantly to EB differentiation downstream of this pathway. Consistent with this notion, our RNA-seq analysis demonstrates that Sox21a regulates a large set of genes including *Pdm1*, which encodes a transcription factor involved in the terminal differentiation of enterocytes^20^. Our study also shows that Sox21a contributes to stem cell division notably in the posterior midgut. This is similar to the JAK/STAT pathway that impacts both stem cell division and differentiation^23^,^24^,^25^. The observation that *Sox21a* flies are viable indicates that the role of Sox21a is likely restricted to the adult intestinal homeostasis. Moving on, functional characterization of Sox21a target genes and identification of Sox21a-binding sites are now required to better understand the role of this transcription factor in ISC proliferation and progenitor differentiation.

An unexpected observation of our work was that *Sox21a* flies developed tumours that increase in size and grow towards the intestinal lumen over time. *Sox21a* tumours are mainly composed of post-mitotic progenitors, the EBs that are blocked in their differentiation. Our study shows that the growth of *Sox21a* tumours relies on ISC division. The requirement for ISC proliferation to drive *Sox21a* tumours explains why tumours are not observed in the posterior midgut and are more frequent when flies are infected with bacteria, a condition that stimulates stem cell proliferation. Our results indicate that the release of a mitogen, Upd2, by accumulating EBs provides a feed-forward loop stimulating ISCs to divide and differentiate, leading to a further increase in the number of EBs. It is likely that *Sox21a* tumours are initiated at random sites where EB clustering leads to a local increase of Upd2. Like other cancer model, *Sox21a* tumours also express matrix metalloproteinase, which probably shapes the tumour local environment to promote tumour progression. Accumulating EBs display a shift in metabolism with an increased expression of ROS-producing factors, such as Duox and a higher number of mitochondria and peroxisomes. This metabolic shift is likely to underlie the increase of ROS at the vicinity of the tumour. We observed that the progenitors express at high-level ROS detoxification enzymes. Thus, the concomitant high-level synthesis of ROS and detoxifying enzymes by accumulating EBs restricts high ROS levels to the tumour borders. It is likely that ROS production promotes *Sox21a* tumour growth by eliminating flanking enterocytes in a JNK-dependent manner. Further experiments are required to determine whether JNK activation in flanking enterocytes is induced by ROS or by mechanical constraints from the tumours or by simultaneously both processes. Our tumour model introduces a new concept highlighting the active role of differentiating stem cell daughters in tumour formation. This model highlights the tumorigenic properties of transit differentiating cells and is in contrast to the current paradigm that emphasizes exclusively on the role of stem cells. In light of our findings, we speculate that the plasticity of these differentiating cells underlies their cancerous properties.

Mechanistic studies of several intestinal tumour models have been reported previously in *Drosophila*^29^,^43^,^51^,^52^,^53^,^54^,^55^. The *Sox21a* tumour model is unique in its simplicity compared with other models that require complex genetic manipulations (for example, *Ras*^*V12*^*Scrib*^*−/−*^ (refs 56, 57). Similar to *Notch* loss-of-function tumour model^43^, it reveals how a differentiation defect in the stem cell progenies can drive tumorigenesis. Both models are caused by a defect in the stem cell differentiation program, rely on stem cell division, and involve the elimination of flanking enterocytes by delamination through the JNK pathway. There are, however, major differences. First, *Notch* tumours are caused by a blockage of differentiation at the ISC stage^14^,^15^,^32^, while *Sox21a* is required later in the differentiation of EBs. This explains their distinct dynamics of tumour formation. Compared with the acute formation of *Notch* ISC tumour, the formation of *Sox21a* EB tumour is a slow and stochastic process. While *Notch* tumours can be observed only a few days after induction of *Notch*-deficient cells, >20 days is required to observe grade 3 tumours in *Sox21a* flies. Second, the growth of *Notch* tumour relies on the autocrine EGF ligand Spitz^43^ and the JAK/STAT ligand Upd2 (our data), while the growth of *Sox21a* tumour requires the paracrine Upd2, and to a much lesser extent Spitz from EBs. Third, *Sox21a* tumours display a higher level of cellular heterogeneity, which has not been described for *Notch* tumours. Fourth, *Sox21a* tumours are not formed in mosaic intestine where *Sox21a* is mutated clonally, while *Notch*-deficient stem cells can grow into tumour in clones. Finally, our study uncovers a non-cell autonomous effect of ROS in tumour progression caused by metabolic changes in the tumour cells. The implication of ROS in cancer is an emerging theme of research. Thus, our model is likely to serve as a useful tool to study how ROS could play a signalling role to mediate short-range communication between tumour cells and their neighbours.

Many human tumours are composed of cells with different degrees of differentiation, including differentiating progenitors derived from stem cells^11^. Our study highlights the cancerous properties of the differentiating stem cell progenies, which can stimulate stem cells proliferation and display a high cellular plasticity. Promoting the terminal differentiation of cancer cells has long been proposed and studied as a promising therapeutic strategy^58^. With increasing knowledge of genetic control of stem cell differentiation, it would be interesting to explore whether modulating progenitor cell differentiation can combat the progression of cancers.

## Methods

### Tumour-burden quantification and statistical analysis

Tumour burden of flies with different genotypes was quantified as follows. Flies were grown either at 25 °C (for mutant analysis) or at 18 °C (for overexpression analysis) until adulthood. Sorted genotypes were kept at 25 °C (for mutant analysis) or at 29 °C (for overexpression analysis) for 20–22 days. The midguts of flies were dissected and scored for tumour burden based on the criteria described below. Grade 0 midguts carried no tumour (Grade 0) and had progenitor cells that were evenly interspersed by large-nucleated enterocytes (Fig. 3g,k). Midguts with cluster of progenitor cells spanning 3–4 enterocytes were designated grade 1 (Fig. 3h,l). Large tumour mass and multilayering cells growing towards the lumen were features of grade 2 and 3 tumours. While enterocytes were still present in grade 1 and 2 tumours, grade 3 tumours were only composed of progenitors. Tumour border was defined as the place where the density of small-nucleated cells reduces to normal. The enterocytes surrounded by the grade 1 and 2 tumour cells were in the process of being eliminated through JNK activation, and we did not count them as part of the tumour. When a fly gut harboured several tumours, we assigned the final grade to the tumour with the highest grade. Overall, 20–30 flies were scored for each experiment and each experiment was repeated for at least three times. The results were pooled to generate graphs of tumour grade distribution in Excel. *P* values were calculated using *χ*^2^-test, and indicated with *P<0.05; **P<0.01; or ***P<0.001. To test the effect of bacterial infection on tumour burden, Gram-negative bacteria *Erwinia carotovora 15* (*Ecc15*) was orally ingested by 7-day-old *Sox21a* flies at OD_600_=100. The flies were returned to normal food after 2 days on *Ecc15*-containing medium, and scored for tumour burden after recovery for another 7 days. Other significance tests in the paper were done with Student's *t*-test using the Prism 5 software.

### *Drosophila* strains

*Drivers*: *esg-Gal4, tub-Gal80^TS^, UAS-GFP* (referred to as *esg^TS^*)^15^; *Myo1A-Gal4, tub-Gal80^TS^, UAS-GFP* (referred to as *Myo1A^TS^*)^23^; *Su(H)GBE-Gal4, UAS-CD8::GFP; tub-Gal80^TS^* (referred to as *GBE^TS^*)^17^; *Su(H)GBE-Gal4, UAS-nlsGFP, tub-Gal80^TS^* (referred to as *GBE^TS^*, this study); *Dl-Gal4, UAS-GFP, tub-Gal80^TS^* (referred to as *Dl^TS^*)^19^; *Act5C-Gal4* (BDSC25374); MARCM tester FRT2A: *y,w,hsFlp; tub-Gal4, UAS-CD8::GFP; FRT2A, tub-Gal80* (gift from Yanrui Jiang) and *y,w,hsFlp, tub-Gal4, UAS-nlsGFP;;FRT2A, tub-Gal80* (this study); MARCM tester FRT82B: *y,w,hsFlp, tub-Gal4, UAS-nlsGFP;;FRT82B, tub-Gal80* (gift from Ernesto Sánchez-Herrero); *GMR43E09-Gal4* (BDSC46247); *puc^E69^-Gal4* (ref. 59), *miR-8-Gal4^NP5247^*(DGRC104917) and *btl-Gal4, UAS-actGFP* (BDSC8807). *Reporters*: *Su(H)-lacZ* (ref. 60); *puc-lacZ^E69^* (DGRC109029); *Dl-lacZ* (gift from Michael Boutros); *gstD-lacZ* (ref. 61); *10xstat-GFP^D^* (ref. 62); *Mmp2-GFP* (BDSC60512); *Dl-GFP* (BDSC59819); *Cat-GFP* (BDSC51546); *sqh-EYFP-Mito* (BDSC7194). *Mutants*: *upd2^Δ^, upd3^Δ^*, and *upd2-3^Δ^* (ref. 63); *FRT82B, Stat^397^* and *hep^1^*. *UAS lines*: *UAS-hop^tumL^* (gift from James Castelli-Gair Hombría); *UAS-Stat-RNAi* (BDSC35600); *UAS-Sox21a-RNAi* (HMJ21395 and HMJ21325); *UAS-N-RNAi* (VDRC100002); *UAS-bsk^DN^* (BDSC6409); *UAS-kay^DN^* (BDSC7214); *UAS-timp, UAS-Mmp1-RNAi*, and *UAS-Mmp2-RNAi* (ref. 38); *UAS-P35* (BDSC5072); *UAS-EGFR^DN^* (BDSC5364); *UAS-GFP.SKL* (BDSC28881), *UAS-mito-HA-GFP* (BDSC8442); *UAS-Sox21a* (this study); *UAS-spi-RNAi* (VDRC103817); *UAS-krn-RNAi* (VDRC104299); *UAS-wg-RNAi* (VDRC13351 and 104579); *UAS-upd1-RNAi* (VDRC3282); *UAS-upd2-RNAi* (BDSC33949 and NIG5988R1-3) and *UAS-upd3-RNAi* (gift from Hervé Agaisse).

The following lines were recombined to *Sox21a*^*6*^ by mitotic recombination: *FRT2A*, *Dl-lacZ*, *Dl-GFP*, *Cat-GFP*, *puc-lacZ*, *puc*^*E69*^*-Gal4*, *tub-Gal80*^*TS*^, *UAS-timp* and *UAS-Mmp2-RNAi.* Full genotypes of flies used in this study are listed in Supplementary Table 1.

In most of our experiments, we used *Sox21a*^*6*^*/+* flies as control. We did not see any difference between *Sox21a*^*6*^*/+* flies and wild-type flies, indicating that *Sox21a*^*6*^ is a recessive mutation.

### Conditional expression of UAS-linked transgenes

The TARGET system was used in combination with the indicated Gal4 drivers to conditionally express UAS-linked transgenes^64^. Flies were grown at 18 °C to limit Gal4 activity. After 3–5 days at 18 °C, adult flies with the appropriate genotypes were shifted to 29 °C, a temperature inactivating the temperature-sensitive Gal80's ability to suppress Gal4.

### MARCM clone induction

The *Sox21a*^*6*^ allele was recombined to *FRT2A* site for MARCM analysis^22^. For clone induction, 3–5-day-old flies with the appropriate genotypes were heat-shocked for 30 min at 37.5 °C in a water bath. The flies were immediately transferred into a new tube and kept at 25 °C until dissection. Rescue experiments were performed by combining the *UAS-Sox21a* transgene with the *FRT2A*, *Sox21a*^*6*^ or *FRT82B*, *Stat*^*397*^ chromosome. Note that *UAS-Sox21a* was only expressed in the clones indicated by the presence of GFP.

### Generation of *Sox21a* mutant and transgenes

*Sox21a* mutant flies were generated with the method described before^21^ with the guide RNA sequence: 5′- *GCTTTCATGGTCTGGTCGCG* -3′. The alleles originally named *Sox21a*^*SK6*^ and *Sox21a*^*SK8*^ were referred to as *Sox21a*^*6*^ and *Sox21a*^*8*^ in the paper.

To generate the enh::*Sox21a* construct, the following primers (5′- *caccATGACGAGCATCTCGGCCCTG* -3′ and 5′- *TCAAATGATGTTTGGCGGACT* -3′) were used to amplify the 2.8-kb *Sox21a-RA*-coding regions together with the intronic *Sox21a* enhancer from the genomic DNA of a wild-type fly. The PCR product was first cloned into pENTR-D-TOPO (Life Technologies) vector, and then swapped into either pTW destination vector to make *UAS-Sox21aEnh::Sox21a* or pTRW destination vector to make *UAS-RFP-Sox21aEnh::Sox21a*. Transgenic flies were established by standard *P* element-mediated germline transformation. At least three independent lines of each construct were tested for expression level. Note that without the presence of a Gal4 activator, the *UAS-RFP-Sox21aEnh::Sox21a* transgene drives the RFP reporter under the control of *Sox21a cis*-regulatory sequence. Despite the presence of RFP, this construct can rescue the *Sox21a* mutation (Fig. 4c).

### Histology and immunostaining

Flies were transferred overnight into a classical fly food vial containing a filter paper soaked with a solution consisting of 5% sucrose to clear the digestive tract. Then, guts of adult females were dissected in PBS, and fixed for at least 1 h at room temperature in 4% paraformaldehyde in PBS. They were subsequently rinsed in PBS+0.1% Triton X-100 (PBT), permeabilized and blocked in 2% bovine serum albumin PBT for 1 h, and incubated with primary antibodies in 2% bovine serum albumin PBT for overnight at 4 °C. After 1-h washing, secondary antibodies and DAPI were applied at room temperature for 2 h. Phalloidin was added to the secondary antibody incubation step in some experiments. Histology and staining on cross-sectioned guts were done as described previously^65^.

For ROS measurement with DHE, guts were dissected and directly incubated in 30 μM DHE (Life Technologies) and DRAQ5 (BioStatus, 1:200) for 10 min at room temperature, washed twice and mounted for confocal microscopy immediately. GFP expressed under the control of *btl>actGFP* was used to identify the location of tumour, and live-cell dye DRAQ5 was used to visualize all the cells.

Primary antibodies used are as follows: mouse anti-Pros (DSHB, 1:100), mouse anti-Arm (DSHB, 1:100), mouse anti-Dlg (DSHB, 1:100), rabbit anti-Pdm1 (1:500, gift from Xiaohang Yang), rabbit anti-pH3 (Millipore, 1:1,000), chicken anti-GFP (Abcam, 1:1,000) and rabbit anti-βGal (Cappel, 1:1,000). Alexa488-, Alexa555- or Alexa647-conjugated secondary antibodies (Life Technologies) were used. Nuclei were counterstained by DAPI (Sigma), and Alexa555 Phalloidin (Life Technologies) was used to stain F-actin. All the images were taken on a Zeiss LSM 700 confocal microscope, except the whole gut on the Olympus slide scanner. Images were processed using Image J and Adobe Photoshop software. Shown in figures are the maximal intensity projections of all the *z*-stacks taken with the confocal, in the same orientation (anterior to the left, and posterior to the right).

### FACS and RNA-seq

Crosses were set-up at 25 °C to obtain *Sox21a/+* (used as control) or homozygous *Sox21a* flies carrying the *GBE>CD8::GFP* transgenes. Eclosed flies were maintained at 25 °C for 16–18 days. Around 150 flies for each biological replicate were dissected in ice-cold 1 × PBS made with diethylpyrocarbonate (DEPC)-treated water under a dry-ice chilled dissecting microscope, within a 1-h time frame. Proventriculus, hindgut and midgut/hindgut junction were removed to collect only midgut *GBE>CD8::GFP*-positive cells. Trimmed midguts were split in the copper cell region of the middle midgut to obtain both the anterior midgut and the posterior midgut. Three biological replicates were performed. Cell dissociation, FACS sorting, total RNA isolation and messenger RNA amplification were performed according to the method described before^33^. Elastase solution with a final concentration of 1 mg ml^−1^ was used for cell dissociation. Cells positive for GFP and negative for propidium iodide were directly sorted into a tube containing RNA extraction buffer on a BD FACSAria II cell sorter. RNA was isolated using the Arcturus PicoPure RNA Isolation Kit (Life Technologies). Messenger RNA was amplified using a two-step linear amplification protocol with the Arcturus RiboAmp HS Plus RNA Amplification Kit (Life Technologies), with 2 ng total RNA as input. We usually obtained >100-μg amplified antisense RNA. Amplified antisense RNA integrity was determined with an Agilent 2100 Bioanalyzer before complementary DNA library preparation with the TruSeq RNA Sample Prep Kit-v2 (Illumina). RNA-seq was performed on a Hi**-**Seq2000 (Illumina) with 100 nt paired**-**end sequencing. RNA-seq data have been deposited in GEO under the accession number GSE71093.

### RNA-seq data analysis

Pair-end reads were mapped to the *Drosophila melanogaster* genome (release version 6.02) using Bowtie2 (ref. 66) with forward and reverse composition. Each sequencing experiment generated in total >40 million raw reads, and >80% was successfully mapped for each experiment. Gene expression was quantified by the number of paired reads that fall into the exons. Normalization was carried out using the size factor formula^67^. Differentially expressed genes were identified using the method as described^68^. Over-represented gene ontology (GO) terms were computed by PANTHER^69^. GO terms were further filtered if the observed number of genes was <5% of the total input number of genes, and 25% of GO terms were discarded according to the ranking of fold enrichment.

### qRT–PCR primers

*Sox21a_F*: 5′- GGACAGAAGCGTCCATTCAT -3′; *Sox21a_R*: 5′- TGACTTGTTGAGCGTCTTGG -3′

*RpL32_F*: 5′- TCTGCATGAGCAGGACCTC -3′; *RpL32_R*: 5′- ATCGGTTACGGATCGAACAA -3′.

## Additional information

**How to cite this article:** Zhai, Z. *et al*. Accumulation of differentiating intestinal stem cell progenies drives tumorigenesis. *Nat. Commun.* 6:10219 doi: 10.1038/ncomms10219 (2015).

## Supplementary Material

Supplementary Information Supplementary Figures 1-10 and Supplementary Table 1
