# Supplementary material for: Accumulation of differentiating intestinal stem cell progenies drives tumorigenesis
Source: Nat Commun. 2015 Dec 22;6:10219. doi: 10.1038/ncomms10219 (PMC4703904; doi:10.1038/ncomms10219)
Supplement: Supplementary Information — Supplementary Figures 1-10 and Supplementary Table 1 [file ncomms10219-s1.pdf]

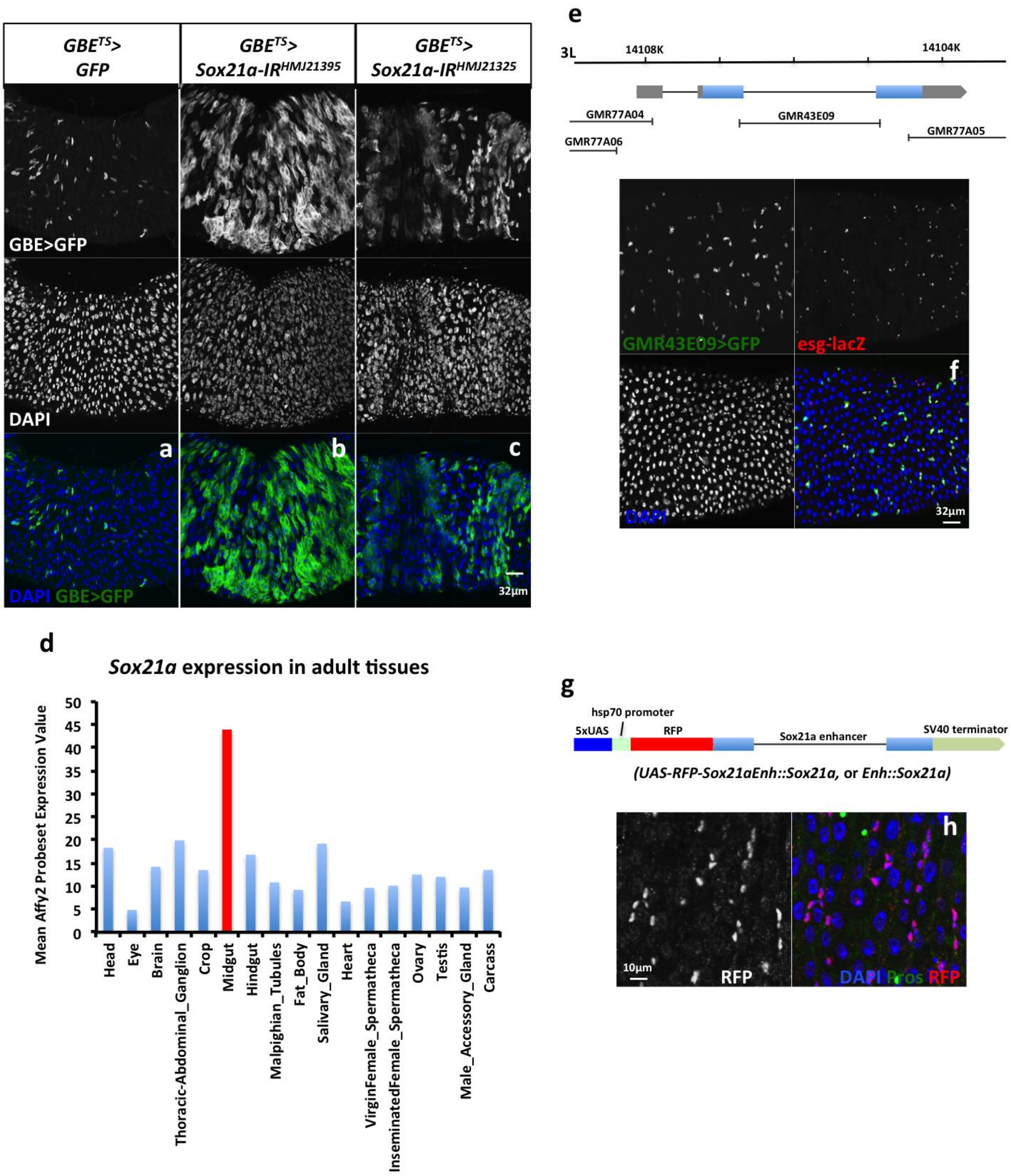

Supplementary Fig. 1. ***Sox21a*** **is a midgut-specific gene involved in EB differentiation**

a-c, Anterior midgut (AMG) of control (a) and 14-day-old flies expressing a *Sox21a* RNAi (b,c) in EB at 29°C. Nuclei are stained for 4',6-diamidino-2-phenylindole (DAPI; middle and bottom row). EB expresses GFP (green, top and bottom row). **d**, Expression profile of *Sox21a* in various adult tissues. Modified from FlyAtlas. **e-f**, Characterization of *Sox21a* enhancers. Of the four putative enhancers shown in e, only *GMR43E09* fragment corresponding to *Sox21a* intron drives *UAS-GFP* expression in the progenitor cells (f). Nuclei are stained with DAPI and progenitors marked by *esg-lacZ*. **g,** Schematic representation of the *Enh::Sox21a-RFP* reporter generated in this study. The enhancer sequence used is the same *GMR43E09* fragment as shown in e. This construct can fully rescue the *Sox21a* mutation (see Fig. 4c). It can be used both for rescue (*Sox21a* is driven by its own enhancer) and for over-expression (due to the presence of UAS element). **h**, the *Enh::Sox21a-RFP* (red) is expressed in all the progenitor cells with a nuclear localization. Pros is co-stained to mark enteroendocrine cells in green. Each image represents 12 flies tested in one experiment (in two biological replicates).

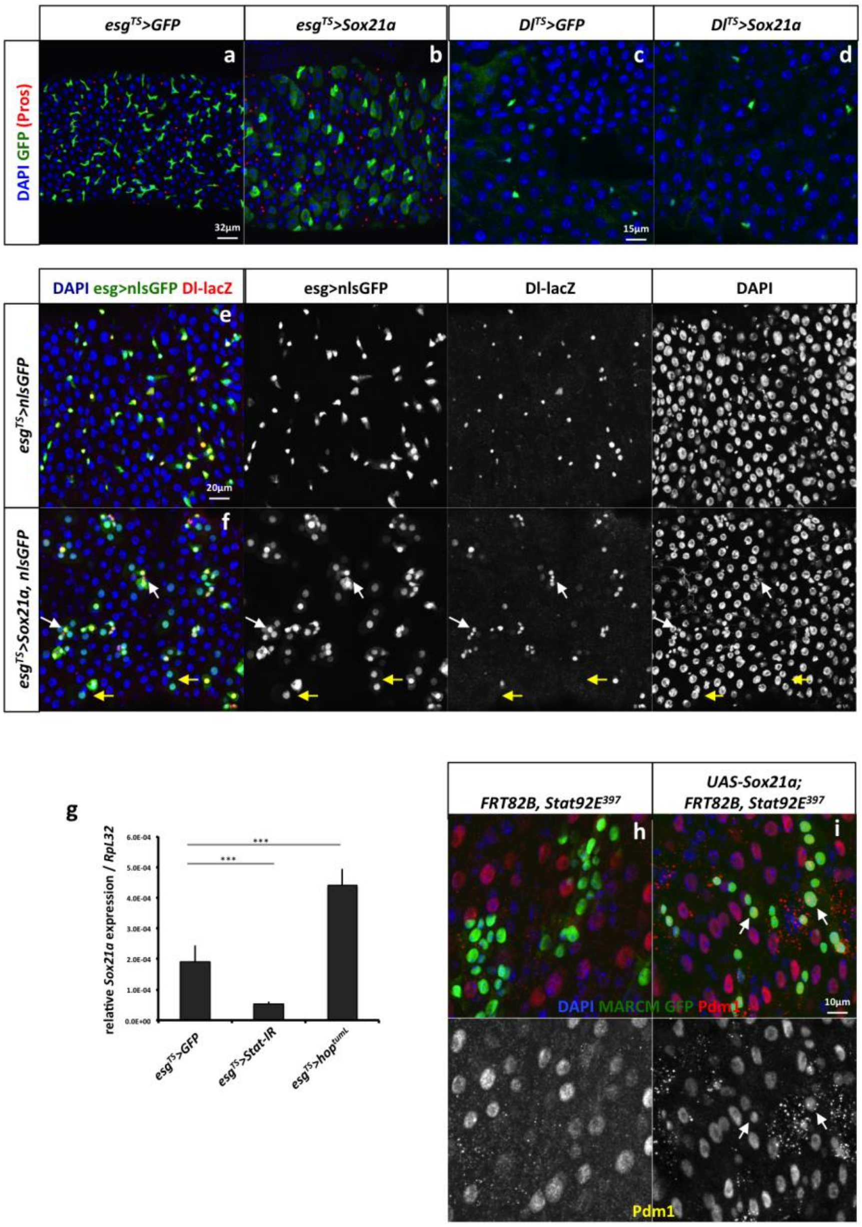

Supplementary Fig. 2. *Sox21a* functions downstream of the JAK/STAT pathway for EB differentiation

a-b, AMG of flies over-expressing GFP (control, a) or *Sox21a* (b) in progenitor cells for 3 days at 29°C using esg^{TS} driver. Enteroendocrine cells are stained in red with the marker Pros. **c-d**, AMG of flies over-expressing GFP (control, c) or *Sox21a* (d) in ISCs for 6 days at 29°C using Dl^{TS} driver. ISCs are shown in green. **e-f**, AMG of control (e) and flies overexpressing *Sox21a* (f) for 4 days in progenitor cells with esg^{TS} using an *UAS-Sox21a* construct with a weak expression level. Over-expression of *Sox21a* induces both progenitor cell differentiation (indicated by yellow arrows in f) and low-level ISC proliferation (indicated by *Dl-lacZ* clusters with white arrows in f). **g**, Relative expression of *Sox21a* mRNA in dissected guts of flies with the indicated genotypes 2 days after shifted to 29°C. Expression is normalized to *RpL32*. hop^{tumL} is a constitutively active form of *Drosophila* JAK hopscotch (hop). **h-i**, GFP positively marked $Stat^{397}$ MARCM clones failed to express the enterocyte marker Pdm1, indicating a defect in differentiation (h). In contrast, some $Stat^{397}$ clone cells over-expressing *Sox21a* specifically in the clone (by a *tub-Gal4*) show expression of Pdm1 (indicated by arrows in i), 6 days after clone induction. *p* values in **g (**4 replicates**)** from

Student's *t*-test (*<0.05; ** <0.01; *** <0.001). Each image represents 12 flies tested in one experiment (in three biological replicates).

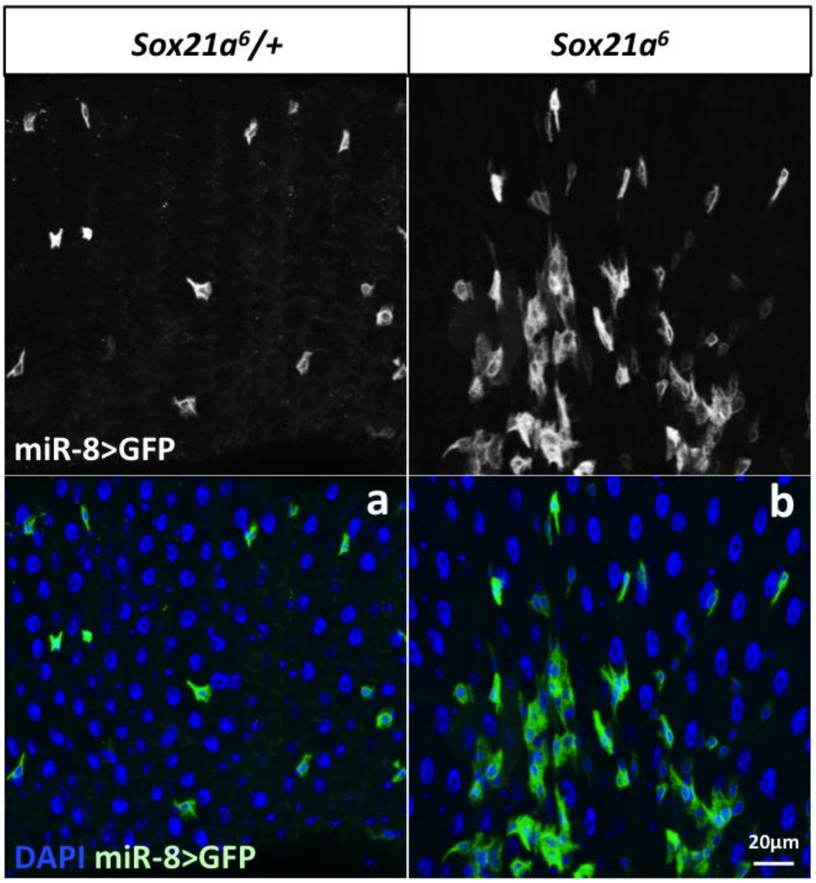

Supplementary Fig. 3. *miR-8* expression in *Sox21a* mutant

a-b, AMG of 10-day-old *Sox21a/+* (control, a) and *Sox21a* flies (b). Nuclei are stained by DAPI (blue). *miR-8* expression is visualized by *miR-8-Gal4 UAS-CD8::GFP* (green). The expression of *miR-8* in progenitors is not affected by the *Sox21a* mutation. Each image represents 12 flies tested in one experiment (in two biological replicates).

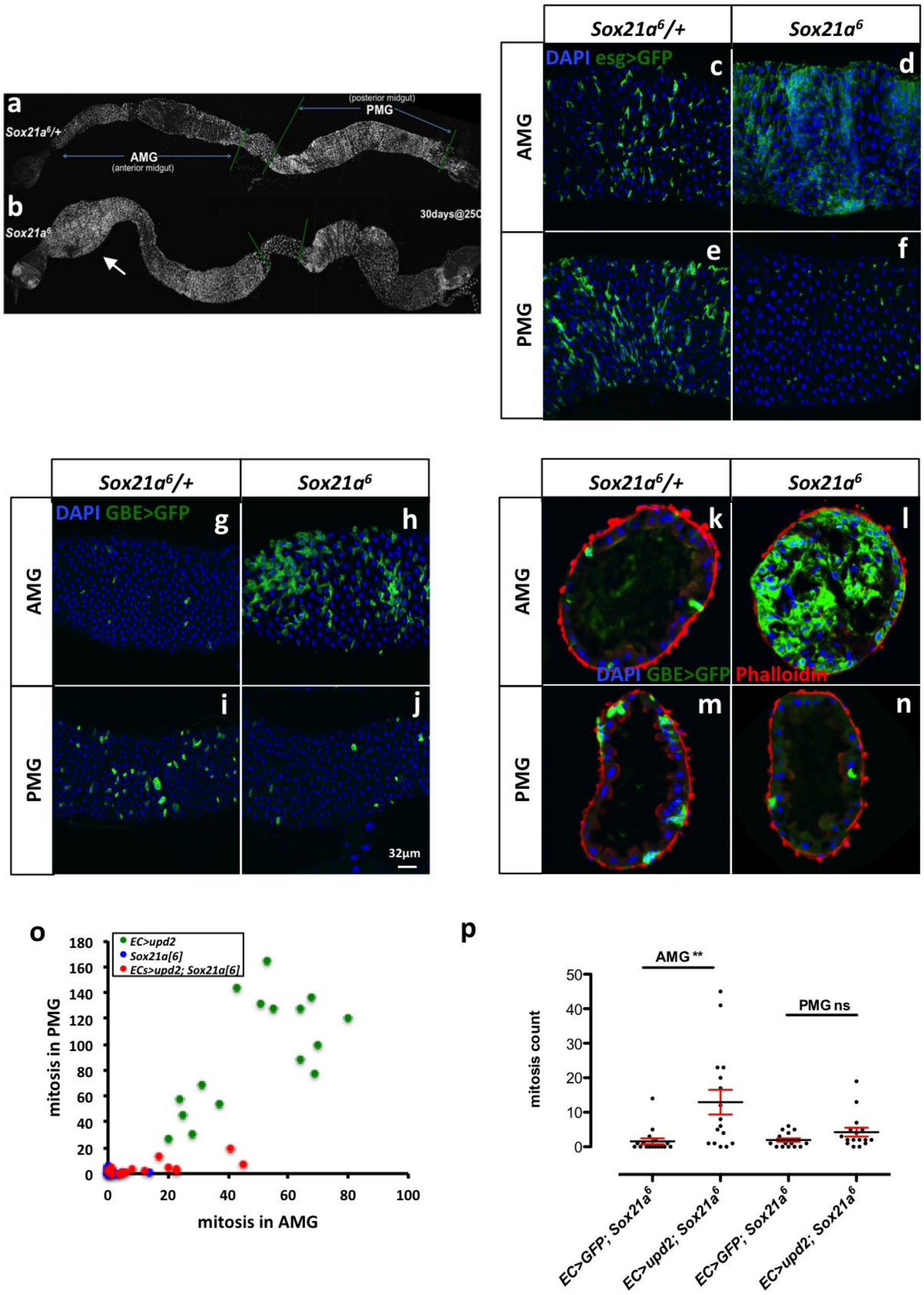

Supplementary Fig. 4. Tumors are found in the anterior midgut of *Sox21a* flies

a-b, The digestive tract of 30-day-old *Sox21a/+* (control, a) and *Sox21a* flies (b) is stained with DAPI. Note the accumulation of cells with small nuclei in the anterior midgut (AMG) but not posterior midgut (PMG) of *Sox21a* flies (arrow). The overall organization of the *Drosophila* gut is preserved. **c-j**, AMG (c, d, g, h) and PMG (e, f, i, j) of 12-day-old *Sox21a/+* (control, c, e, g, i) and *Sox21a* flies (d, f, h, j). Nuclei are stained by DAPI (blue). Progenitors (*esg>GFP)* (c-f) and EBs (*GBE>GFP)* (g-j) are visualized in green. **k-n**, Cross-sections of AMG or PMG of 21-day-old *Sox21a/+* (control, k, m) and *Sox21a* flies (l, n). Phalloidin binds to F-actin to mark the visceral muscles that surround the intestine (red). EBs are in green. **o-p**, mitoses in both AMG and PMG of each 7-day-old flies with indicated genotypes after being shifted to 29°C to induce Upd2 expression for 3 days (n=16, in 2 replicates). Error bars indicate s.e.m. *p* values in **p** from Student's *t*-test

(*<0.05; ** <0.01; *** <0.001; ns: not significant). Each image represents 12 flies tested in one experiment (in three biological replicates).

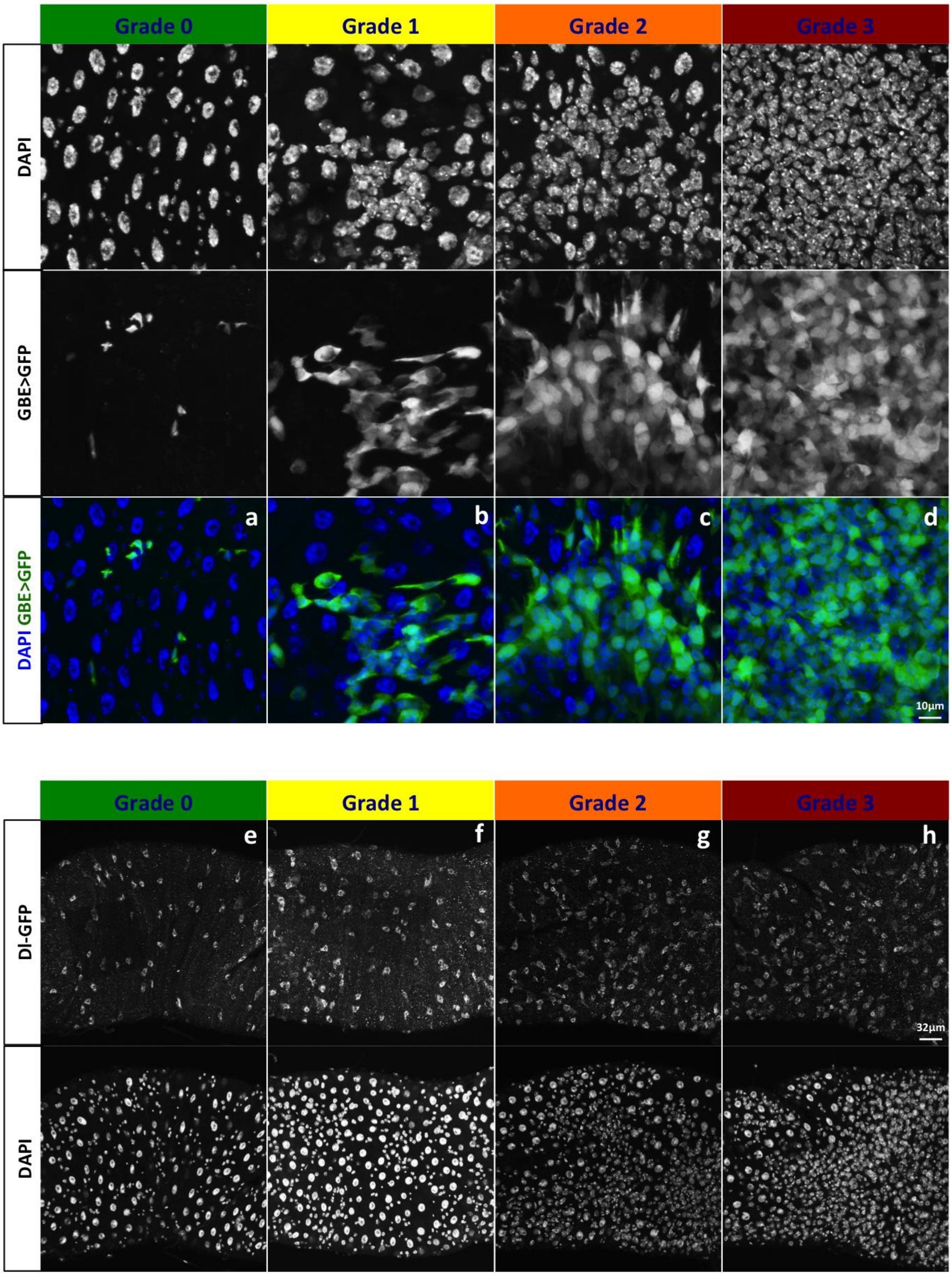

Supplementary Fig. 5. *Sox21a* flies harbor tumor of different grades.

a-h, Representative images of tumors found in *Sox21a* flies. EBs are marked by *GBE>nlsGFP* (a-d). ISCs are marked by *Dl-GFP* (e-h). Enterocytes can be recognized by their big nuclei size (DAPI). Tumor grades are indicated at the top of each panel. Tumors vary in size and degree of overgrowth. DAPI staining reveals the progressive loss of enterocytes around the tumor site. Each image represents 16 flies tested in one experiment (in three biological replicates).

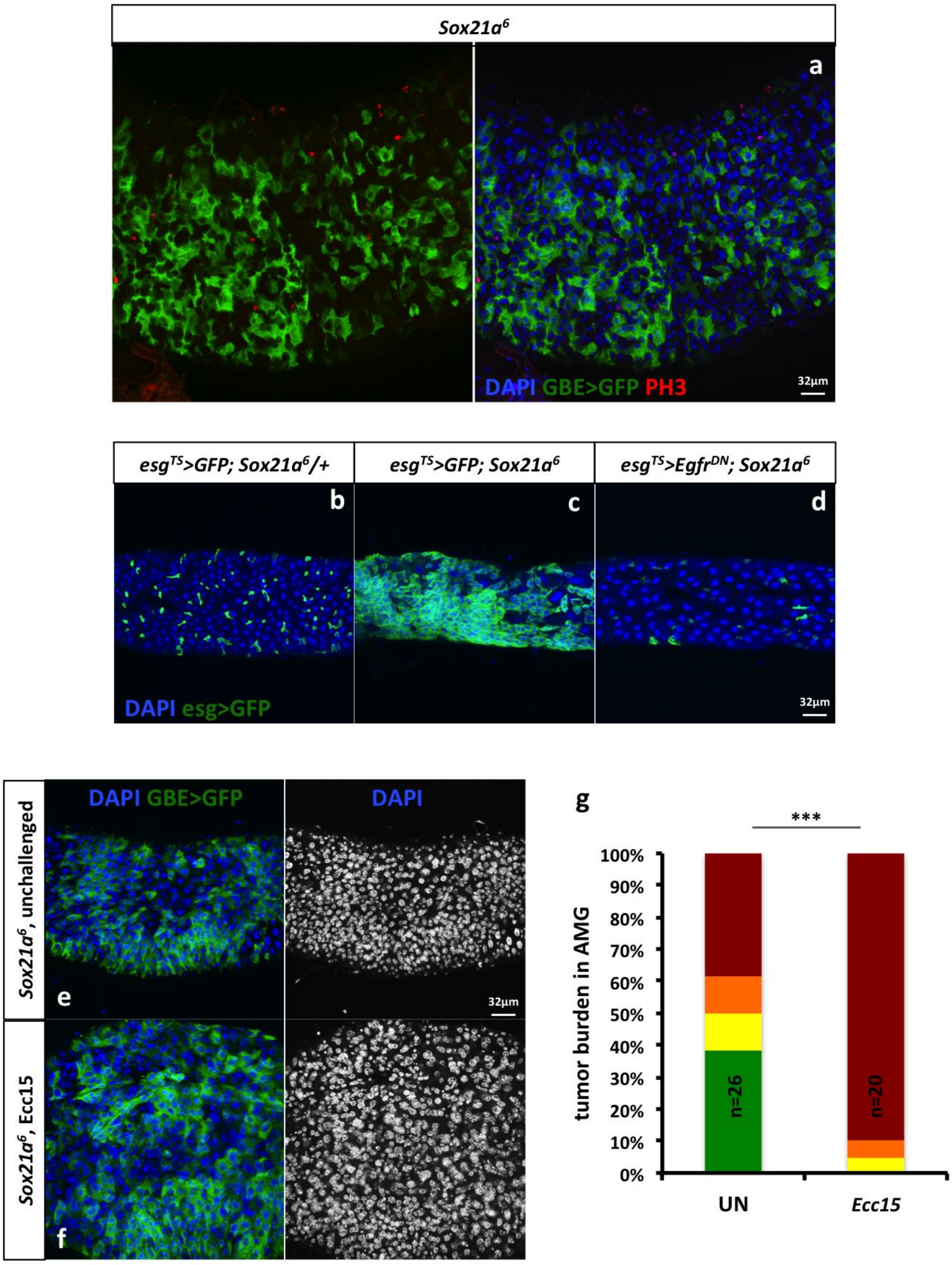

Supplementary Fig. 6. Tumor formation of *Sox21a* flies depends on ISC proliferation

a, AMG of 16-day-old *Sox21a* flies stained for EB marker *GBE>GFP* (green) and a mitotic marker phospho-Histone H3 (PH3, red). None of the PH3 positive cells expresses EB marker. **b-d**, AMG of *Sox21a/+* flies (control, b), *Sox21a* flies (c), and *Sox21a* flies expressing a dominant-negative form of EGFR specifically in progenitor cells (d) for 21 days at 29°C. Nuclei are stained by DAPI (blue). Progenitors are marked by *esg>GFP*. **e-g**, representative images (e-f) and quantification (g) of tumor burden in *Sox21a* flies of unchallenged control (e, g) and *Ecc15* infection (f, g). 7-day-old *Sox21a* flies were treated for 2 days with *Ecc15* and recovered for another 10 days before

dissection. *p* value from Chi-square test (*<0.05; ** <0.01; *** <0.001). Each image represents 20 flies tested in one experiment (in three biological replicates).

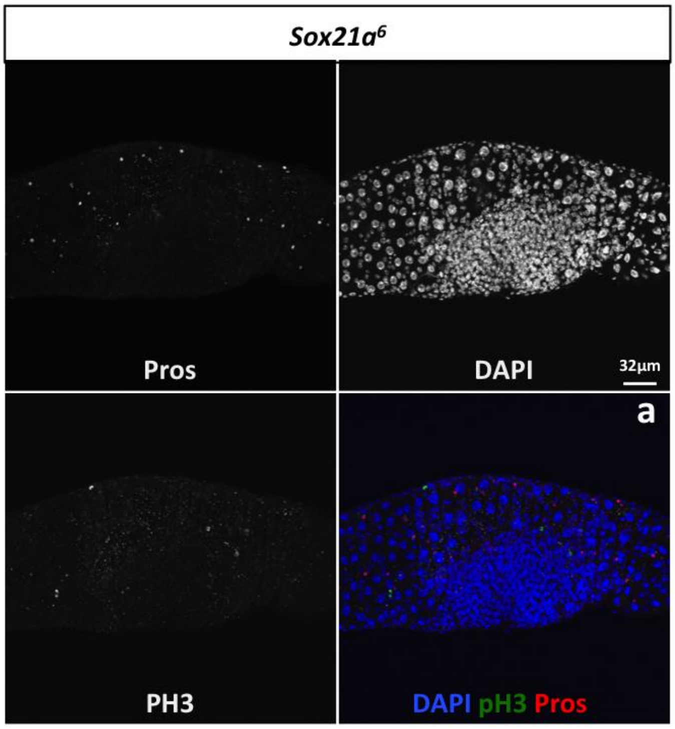

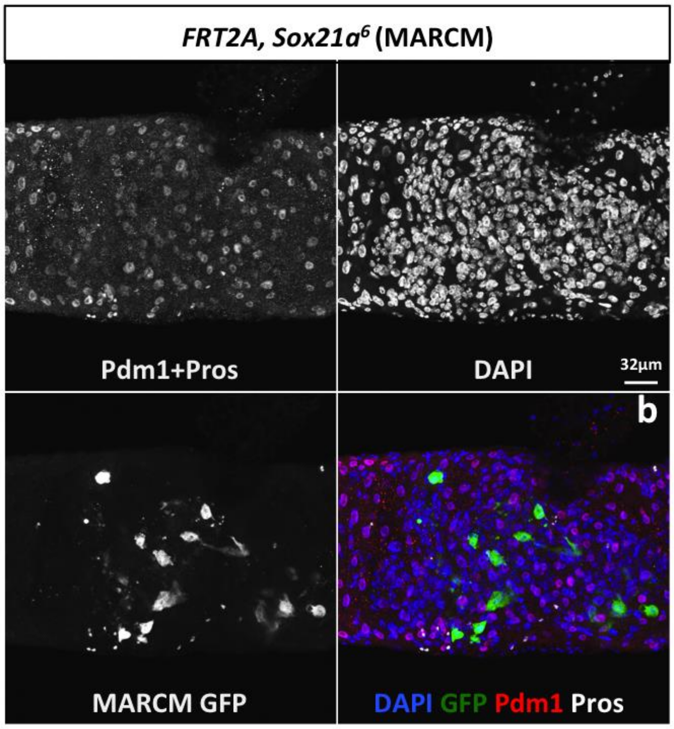

Supplementary Fig. 7. *Sox21a* tumor cells induce non-cell autonomous ISC growth

a, A tumor from the AMG of a 2-week-old *Sox21a* flies stained for enteroendocrine cell marker (Pros), a mitotic marker (PH3) and DAPI. *Sox21a* tumor does not contain any enteroendocrine cell. Note the higher number of mitotic cells around the tumor. **b**, GFP positively marked *Sox21a* MARCM clones at 21 days after clone induction. Pdm1 and Pros mark the differentiated cells. The DAPI staining reveals a local and non-cell autonomous hyperplasia around *Sox21a* mutant cells.

Cells surrounding the clone are not blocked in their differentiation. Each image represents 15 flies tested in one experiment (in three biological replicates).

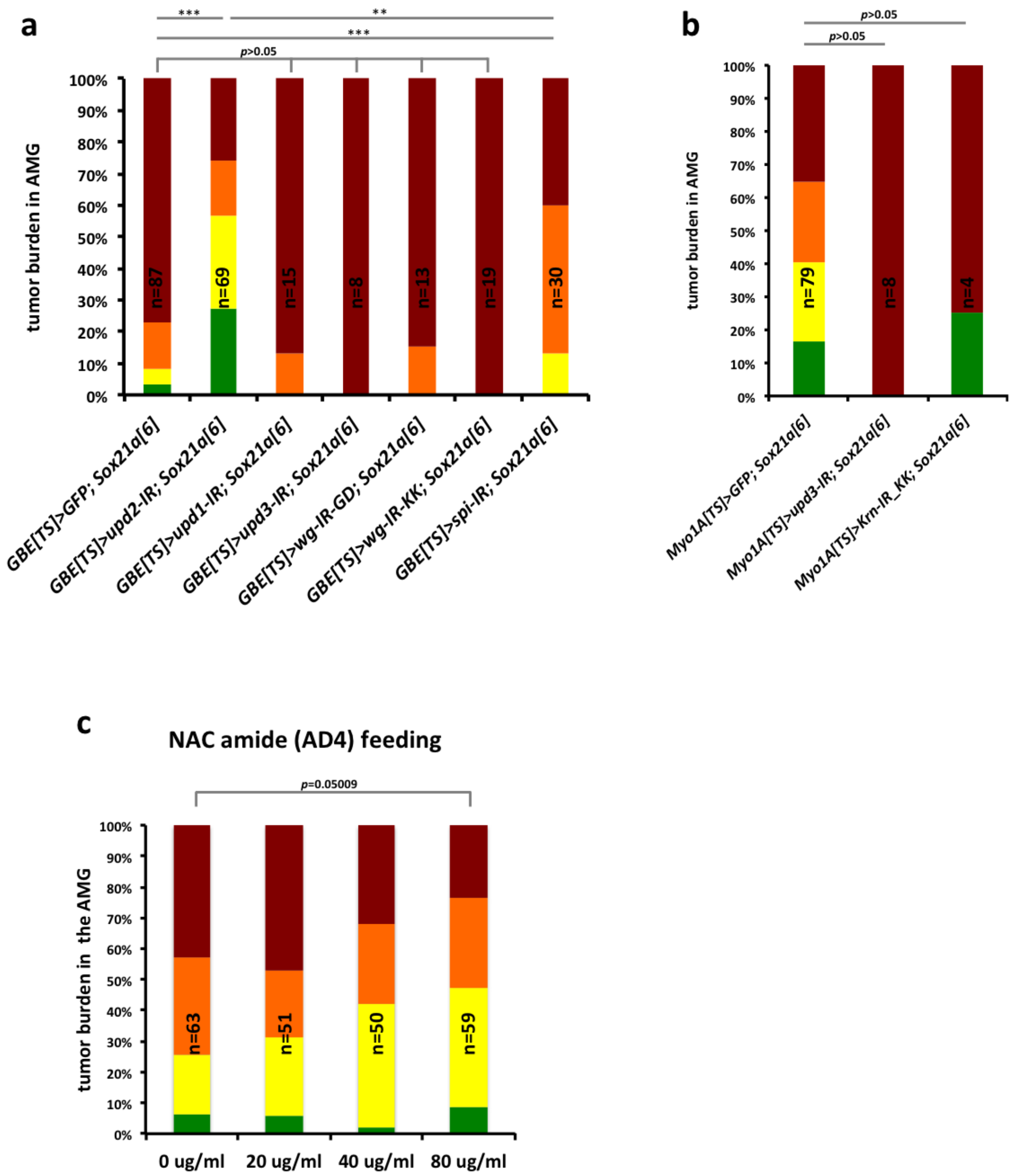

Supplementary Fig. 8. Expression of Upd2 by EBs is required for tumor growth

a-b, Shown are the tumor burdens in the AMG of flies with the indicated genotypes. Flies were raised for 21 days at 29°C. Silencing *upd2* (and to a much lesser extent the EGF *spitz*) in EBs reduced the tumor burden (a). Silencing *upd3* or the EGFR ligand-encoding gene *krn* in enterocytes did not reduce the tumor burden of *Sox21a* flies (b). **c**, Tumor burden in the AMG of *Sox21a* flies fed with increasing concentration of NAC amide (AD4) for 3 weeks. *p* values from Chi-square test (*<0.05; ** <0.01; *** <0.001). Numbers of flies scored for each genotype are indicated. Results in **a-b** represent three independent experiments, and **c** was repeated twice.

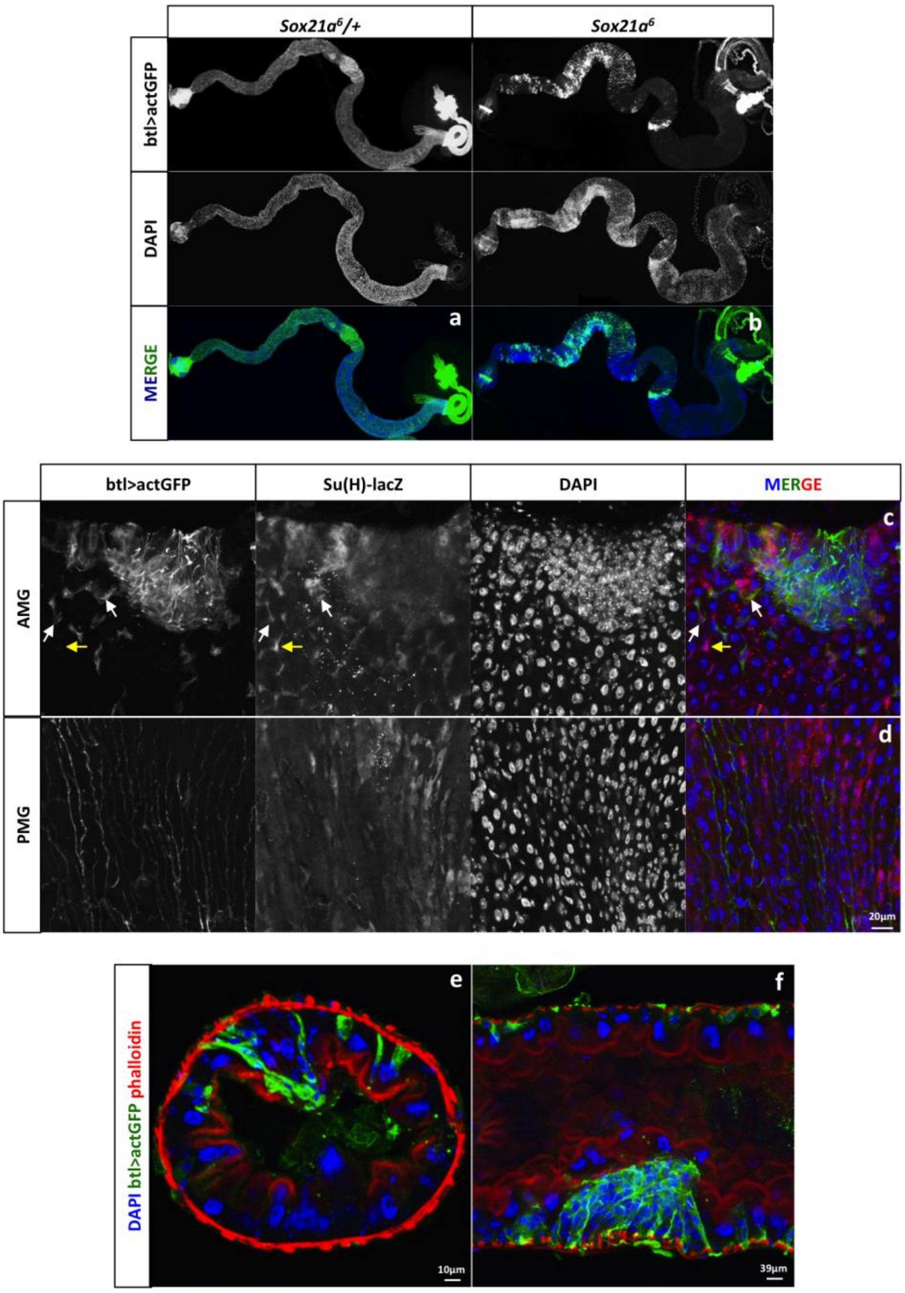

Supplementary Fig. 9. Some *Sox21a* EBs express *btl>actGFP*

a-b, Overview of midgut of 3 week-old *Sox21a/+* (control, a) and *Sox21a* (b) flies. *breathless* (*btl*) expression is visualized by *btl>actGFP* in green. Note that the midgut cells do not normally express *btl>actGFP* (a). In contrast, strong expression of *btl>actGFP* was found at the site of tumor in the anterior midgut (AMG) but not the posterior midgut (PMG) of *Sox21a* flies (b). Tumors can be distinguished by the high density of DAPI signal due to the accumulation of small-nucleated cells (b). **c-d**, High magnification of a tumor from 10-day-old *Sox21a* flies shows the presence of *btl>actGFP* expressing cells at the site of tumor (AMG, top panels). Presence of *btl>actGFP* expressing cell inside the intestinal epithelium was not observed in the PMG (lower panels). Examples of cells both positive for *btl>actGFP* and the EB marker *Su(H)-lacZ* are indicated by white arrows. Note that some EBs do not express *btl>actGFP* (indicated by yellow arrow). **e-f**, A cross-section (e) and a confocal section (f) of *Sox21a* AMG of a 14-day-old fly expressing *btl>actGFP* and co-stained for phalloidin in red. Each image represents 20 flies tested in one experiment (in three biological replicates).

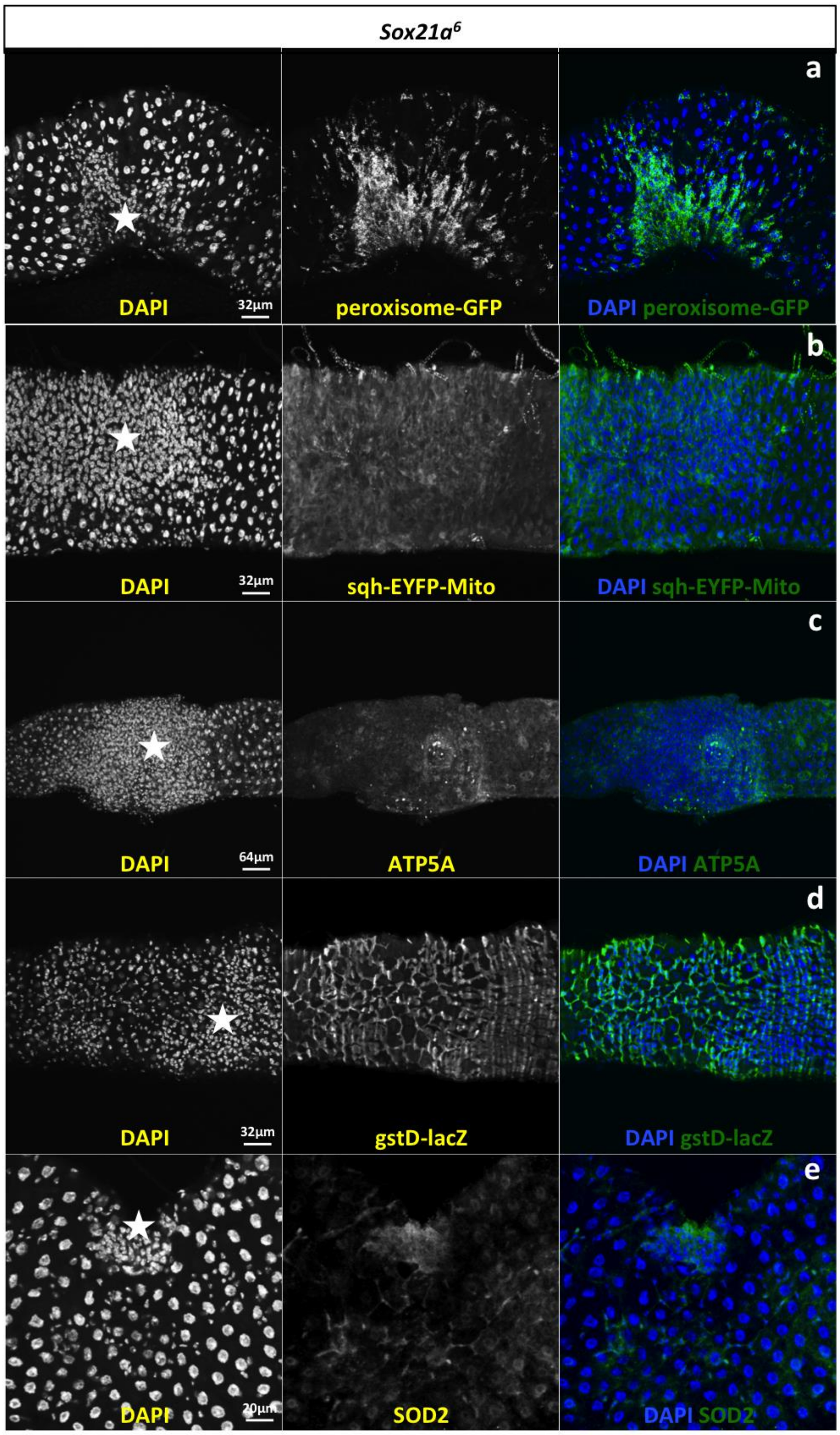

Supplementary Fig. 10. Tumors of *Sox21a* flies modulate ROS metabolism

a-e, Intestines of *Sox21a* flies with tumor stained for DAPI (left panel) and different markers (middle panel). The position of the tumor composed of a cluster of small-nucleated cells is indicated by a star (left panel). The merge of DAPI and different markers is shown in right panel. *Peroxisome-GFP*, *sqh-EYFP-mito*, and *gstD-lacZ* are reporter genes for peroxisomes, mitochondria, and *glutathione S transferase D1* (*gstD*) that encodes an anti-oxidant protein, respectively. The presence of ATP5A, a mitochondrial membrane ATP synthase, and Superoxide dismutase 2 (SOD2) was detected by immunostaining. Each image represents 20 flies tested in one experiment (in three biological replicates).

Supplementary Table 1 Full genotypes of flies used in this study

**Figure 1**	
**b**	*w^{-}; Su(H)GBE-Gal4, UAS-CD8::GFP/+; tub-Gal80^{TS}/+*
**c**	*w^{-}; Su(H)GBE-Gal4, UAS-CD8::GFP/UAS-Sox21a-RNAi^{HMJ21395}; tub-Gal80^{TS}/+*
**e**	*ywhsFlp, tub-Gal4, UAS-nlsGFP/+; +/+; FRT2A, tub-Gal80/FRT2A*
**f**	*ywhsFlp, tub-Gal4, UAS-nlsGFP/+; +/+; FRT2A, tub-Gal80/ FRT2A, Sox21a^{6}*
**g**	*ywhsFlp, tub-Gal4, UAS-nlsGFP/+; UAS-Sox21a/+; FRT2A, tub-Gal80/ FRT2A, Sox21a^{6}*
**Figure 2**	
**a**	*w^{-}; esg-Gal4, UAS-CD8::GFP, tub-Gal80^{TS}/+; +/+*
**b**	*w^{-}; esg-Gal4, UAS-CD8::GFP, tub-Gal80^{TS}/+; UAS-Sox21a/+*
**d**	*w^{-}; Su(H)GBE-Gal4, UAS-CD8::GFP /+; +/ tub-Gal80^{TS}*
**e**	*w^{-}; Su(H)GBE-Gal4, UAS-CD8::GFP /+; UAS-Sox21a/ tub-Gal80^{TS}*
**Figure 3**	
**a**	*w^{-}; esg-Gal4, UAS-CD8::GFP, tub-Gal80^{TS}/+; Sox21a^{6}/+*
**b**	*w^{-}; esg-Gal4, UAS-CD8::GFP, tub-Gal80^{TS}/+; Sox21a^{6}/FRT2A, Sox21a^{6}*
**c**	*w^{-}; Su(H)GBE-Gal4, UAS-CD8::GFP /+; Sox21a^{6}, Dl-lacZ/+*
**d-e**	*w^{-}; Su(H)GBE-Gal4, UAS-CD8::GFP /+; Sox21a^{6}, Dl-lacZ /FRT2A, Sox21a^{6}*
**f, g-j**	*w^{-}; +/+; FRT2A, Sox21a^{6}*
**k-n**	*w^{-}; Su(H)GBE-Gal4, UAS-CD8::GFP /+; Sox21a^{6} /FRT2A, Sox21a^{6}*
**o**	*w^{-}; +/+; Dl-GFP, Sox21a^{6}/ FRT2A, Sox21a^{6}*
**p**	*w^{-}; Su(H)GBE-Gal4, UAS-nlsGFP, tub-Gal80^{TS}/+; Sox21a^{6}/ FRT2A, Sox21a^{6}*
**Figure 4**	
**a**	*w^{-}; 10xStat-GFP^{D}/+; FRT2A, Sox21a^{6}*
**b**	*ywhsFlp/+; tub-Gal4, UAS-CD8::GFP/+; FRT2A, tub-Gal80/ FRT2A, Sox21a^{6}*
**c**	*w^{-}; +/+; FRT2A, Sox21a^{6}*
	w^{-}; UAS-RFP-Sox21aEnh::Sox21a/+; FRT2A, Sox21a^{6}
	upd2-3$^{\Delta}$/upd2-3$^{\Delta}$; +/+; FRT2A, Sox21a^{6}
	upd2$^{\Delta}$/upd2$^{\Delta}$; +/+; FRT2A, Sox21a^{6}
	upd3$^{\Delta}$/upd3$^{\Delta}$; +/+; FRT2A, Sox21a^{6}
	w^{-}; Su(H)GBE-Gal4, UAS-nlsGFP, tub-Gal80^{TS}/+; Sox21a^{6}/ FRT2A, Sox21a^{6}
	w^{-}; Su(H)GBE-Gal4, UAS-nlsGFP, tub-Gal80^{TS}/UAS-upd2-RNAi^{NIG}; Sox21a^{6}/ FRT2A, Sox21a^{6}
	w^{-}; MyolA-Gal4, UAS-nlsGFP, tub-Gal80^{TS}/+; Sox21a^{6}/ FRT2A, Sox21a^{6}
	w^{-}; MyolA-Gal4, UAS-nlsGFP, tub-Gal80^{TS}/UAS-upd2-RNAi^{NIG}; Sox21a^{6}/ FRT2A, Sox21a^{6}
**d**	*w^{-}; Su(H)GBE-Gal4, UAS-nlsGFP, tub-Gal80^{TS}/+; Sox21a^{6}/ FRT2A, Sox21a^{6}*
**e**	*w^{-}; Su(H)GBE-Gal4, UAS-nlsGFP, tub-Gal80^{TS}/UAS-upd2-RNAi^{NIG}; Sox21a^{6}/ FRT2A, Sox21a^{6}*
**f**	*w^{-}; esg-Gal4, UAS-CD8::GFP, tub-Gal80^{TS}/UAS-N-RNAi^{KK}; +/+*
**g**	*w^{-}; esg-Gal4, UAS-CD8::GFP, tub-Gal80^{TS}/UAS-N-RNAi^{KK}; UAS-upd2-RNAi^{BL33949}/+*
**h**	*w^{-}; esg-Gal4, UAS-CD8::GFP, tub-Gal80^{TS}/+; +/+*
**i**	*w^{-}; esg-Gal4, UAS-CD8::GFP, tub-Gal80^{TS}/+; UAS-upd2-RNAi^{BL33949}/+*
**Figure 5**	
**a**	*w^{-}; Su(H)GBE-Gal4, UAS-CD8::GFP /+; Sox21a^{6}/+*
	w^{-}; Su(H)GBE-Gal4, UAS-CD8::GFP /+; Sox21a^{6}/FRT2A, Sox21a^{6}
**Figure 6**	

**a**	*w*$^{-}$*; Mmp2-GFP/+; FRT2A, Sox21a*^{6}*/+*
**b**	*w*$^{-}$*; Mmp2-GFP/+; FRT2A, Sox21a*^{6}
**c**	*w*$^{-}$*; Su(H)GBE-Gal4, UAS-nlsGFP, tub-Gal80*^{TS}*/+; Sox21a*^{6}*/ FRT2A, Sox21a*^{6}
**d**	*w*$^{-}$*/UAS-bsk*^{DN}*; Su(H)GBE-Gal4, UAS-nlsGFP, tub-Gal80*^{TS}*/+; Sox21a*^{6}*/ FRT2A, Sox21a*^{6}
**e**	*w*$^{-}$*; Su(H)GBE-Gal4, UAS-nlsGFP, tub-Gal80*^{TS}*/+; Sox21a*^{6}*, UAS-Mmp2-RNAi*$^{M.U.}$*/ FRT2A, Sox21a*^{6}
**f**	*w*$^{-}$*; Su(H)GBE-Gal4, UAS-nlsGFP, tub-Gal80*^{TS}*/+; Sox21a*^{6}*/ FRT2A, Sox21a*^{6}
	w^{-}*/UAS-bsk*^{DN}*; Su(H)GBE-Gal4, UAS-nlsGFP, tub-Gal80*^{TS}*/+; Sox21a*^{6}*/ FRT2A, Sox21a*^{6}
	w^{-}*; Su(H)GBE-Gal4, UAS-nlsGFP, tub-Gal80*^{TS}*/UAS-kay*^{DN}*; Sox21a*^{6}*/ FRT2A, Sox21a*^{6}
	w^{-}*; Su(H)GBE-Gal4, UAS-nlsGFP, tub-Gal80*^{TS}*/+; Sox21a*^{6}*, UAS-timp/ FRT2A, Sox21a*^{6}
	w^{-}*; Su(H)GBE-Gal4, UAS-nlsGFP, tub-Gal80*^{TS}*/+; Sox21a*^{6}*, UAS-Mmp2-RNAi*$^{M.U.}$*/ FRT2A, Sox21a*^{6}
	w^{-}*; Su(H)GBE-Gal4, UAS-nlsGFP, tub-Gal80*^{TS}*/UAS-Mmp1-RNAi*$^{M.U.}$*; Sox21a*^{6}*/ FRT2A, Sox21a*^{6}
**Figure 7**	
**a**	*w*$^{-}$*; UAS-CD8::GFP/+; puc*^{E69}*-Gal4, Sox21a*^{6}*/FRT2A, Sox21a*^{6}
**b-d**	*w*$^{-}$*; Su(H)GBE-Gal4, UAS-CD8::GFP/UAS-Sox21a-RNAi*^{HMJ21395}*; tub-Gal80*^{TS}*/puc*^{E69}*-lacZ*
**e**	*w*$^{-}$*; +/+; Sox21a*^{6}*/FRT2A, Sox21a*^{6}
	w^{-}*; +/+; Sox21a*^{6}*, puc*^{E69}*-lacZ /FRT2A, Sox21a*^{6}
	w^{-}*/hep*^{1}*; +/+; Sox21a*^{6}*/FRT2A, Sox21a*^{6}
	w^{-}*; MyoIA-Gal4, UAS-nlsGFP, tub-Gal80*^{TS}*/+; Sox21a*^{6}*/ FRT2A, Sox21a*^{6}
	w^{-}*/UAS-bsk*^{DN}*; MyoIA-Gal4, UAS-nlsGFP, tub-Gal80*^{TS}*/+; Sox21a*^{6}*/ FRT2A, Sox21a*^{6}
	w^{-}*; MyoIA-Gal4, UAS-nlsGFP, tub-Gal80*^{TS}*/UAS-p35; Sox21a*^{6}*/ FRT2A, Sox21a*^{6}
**Figure 8**	
**a**	*w*$^{-}$*; Act5C-Gal4/UAS-mito-HA-GFP; Sox21a*^{6}*, tub-Gal80*^{TS}*/+*
**b**	*w*$^{-}$*; Act5C-Gal4/UAS-mito-HA-GFP; Sox21a*^{6}*, tub-Gal80*^{TS}*/ FRT2A, Sox21a*^{6}
**c**	*w*$^{-}$*; Act5C-Gal4/UAS-GFP.SKL; Sox21a*^{6}*, tub-Gal80*^{TS}*/+*
**d-e**	*w*$^{-}$*; Act5C-Gal4/UAS-GFP.SKL; Sox21a*^{6}*, tub-Gal80*^{TS}*/ FRT2A, Sox21a*^{6}
**f**	*w*$^{-}$*/Su(H)-lacZ; +/+; FRT2A, Sox21a*^{6} */Cat-GFP*
**g-h**	*w*$^{-}$*/Su(H)-lacZ; +/+; FRT2A, Sox21a*^{6} */ Cat-GFP, Sox21a*^{6}
**i**	*w*$^{-}$*; btl-Gal4, UAS-actGFP/+; FRT2A, Sox21a*^{6}
**j**	*w*$^{-}$*; Su(H)GBE-Gal4, UAS-nlsGFP, tub-Gal80*^{TS}*/+; Sox21a*^{6}*, puc*^{E69}*-lacZ/+*
**k**	*w*$^{-}$*; Su(H)GBE-Gal4, UAS-nlsGFP, tub-Gal80*^{TS}*/UAS-Duox; Sox21a*^{6}*, puc*^{E69}*-lacZ/+*
**Supplementary Figure 1**	
**a**	*w*$^{-}$*; Su(H)GBE-Gal4, UAS-CD8::GFP/+; tub-Gal80*^{TS}
**b**	*w*$^{-}$*; Su(H)GBE-Gal4, UAS-CD8::GFP/UAS-Sox21a-RNAi*^{HMJ21395}*; tub-Gal80*^{TS}
**c**	*w*$^{-}$*; Su(H)GBE-Gal4, UAS-CD8::GFP/UAS-Sox21a-RNAi*^{HMJ21325}*; tub-Gal80*^{TS}
**f**	*w*$^{-}$*; esg-lacZ/+; GMR43E09-Gal4, UAS-CD8::GFP/+*
**h**	*w*$^{-}$*; ; UAS-RFP-Sox21aEnh::Sox21a*
**Supplementary Figure 2**	
**a**	*w*$^{-}$*; esg-Gal4, UAS-CD8::GFP, tub-Gal80*^{TS}*/+; +/+*
**b**	*w*$^{-}$*; esg-Gal4, UAS-CD8::GFP, tub-Gal80*^{TS}*/+; UAS-Sox21a/+*

**c**	w^{-}; tub-Gal80^{TS}/+; Dl-Gal4, UAS-GFP/+
**d**	w^{-}; tub-Gal80^{TS}/+; Dl-Gal4, UAS-GFP/UAS-Sox21a
**e**	w^{-}; esg-Gal4, UAS-nlsGFP, tub-Gal80^{TS}/+; Dl-lacZ/+
**f**	w^{-}; esg-Gal4, UAS-nlsGFP, tub-Gal80^{TS}/UAS-Sox21a; Dl-lacZ/+
**g**	w^{-}; esg-Gal4, UAS-CD8::GFP, tub-Gal80^{TS}/+; +/+
	w^{-}; esg-Gal4, UAS-CD8::GFP, tub-Gal80^{TS}/UAS-Stat-RNAi; +/+
	w^{-}; esg-Gal4, UAS-CD8::GFP, tub-Gal80^{TS}/UAS-hop^{tumL}; +/+
**h**	ywhsFlp, tub-Gal4, UAS-nlsGFP/+; +/+; FRT82B, tub-Gal80/ FRT82B, Stat^{397}
**i**	ywhsFlp, tub-Gal4, UAS-nlsGFP/+; UAS-Sox21a/+; FRT82B, tub-Gal80/ FRT82B, Stat^{397}
**Supplementary Figure 3**	
**a**	w^{-}; miR-8-Gal4/ UAS-CD8::GFP; FRT2A, Sox21a^{6}/+
**b**	w^{-}; miR-8-Gal4/ UAS-CD8::GFP; FRT2A, Sox21a^{6}
**Supplementary Figure 4**	
**a**	w^{-}; +/+; Sox21a^{6}/+
**b**	w^{-}; +/+; Sox21a^{6}
**c,e**	w^{-}; esg-Gal4, UAS-CD8::GFP, tub-Gal80^{TS}/+; Sox21a^{6}/+
**d,f**	w^{-}; esg-Gal4, UAS-CD8::GFP, tub-Gal80^{TS}/+; Sox21a^{6}/FRT2A, Sox21a^{6}
**g,i,k,m**	w^{-}; Su(H)GBE-Gal4, UAS-CD8::GFP /+; Sox21a^{6}/+
**h,j,l,n**	w^{-}; Su(H)GBE-Gal4, UAS-CD8::GFP /+; Sox21a^{6}/ FRT2A, Sox21a^{6}
**o,p**	w^{-}; MyolA-Gal4, UAS-nlsGFP, tub-Gal80^{TS}/ UAS-upd2GFP; +/+
	w^{-}; MyolA-Gal4, UAS-nlsGFP, tub-Gal80^{TS}/+; Sox21a^{6}/ FRT2A, Sox21a^{6}
	w^{-}; MyolA-Gal4, UAS-nlsGFP, tub-Gal80^{TS}/UAS-upd2.GFP; Sox21a^{6}/ FRT2A, Sox21a^{6}
**Supplementary Figure 5**	
**a-d**	w^{-}; Su(H)GBE-Gal4, UAS-nlsGFP, tub-Gal80^{TS}/+; Sox21a^{6}/ FRT2A, Sox21a^{6}
**e-h**	w^{-}; +/+; Dl-GFP, Sox21a^{6}/ FRT2A, Sox21a^{6}
**Supplementary Figure 6**	
**a**	w^{-}; Su(H)GBE-Gal4, UAS-CD8::GFP /+; Sox21a^{6}/ FRT2A, Sox21a^{6}
**b**	w^{-}; esg-Gal4, UAS-CD8::GFP, tub-Gal80^{TS}/+; Sox21a^{6}/+
**c**	w^{-}; esg-Gal4, UAS-CD8::GFP, tub-Gal80^{TS}/+; Sox21a^{6}/FRT2A, Sox21a^{6}
**d**	w^{-}; esg-Gal4, UAS-CD8::GFP, tub-Gal80^{TS}/ UAS-Egfr^{DN}; Sox21a^{6}/FRT2A, Sox21a^{6}
**e-g**	w^{-}; Su(H)GBE-Gal4, UAS-CD8::GFP /+; Sox21a^{6}/ FRT2A, Sox21a^{6}
**Supplementary Figure 7**	
**a**	w^{-}; +/+; Sox21a^{6}
**b**	ywhsFlp/+; tub-Gal4, UAS-CD8::GFP/+; FRT2A, tub-Gal80/ FRT2A, Sox21a^{6}
**Supplementary Figure 8**	
**a**	w^{-}; Su(H)GBE-Gal4, UAS-nlsGFP, tub-Gal80^{TS}/+; Sox21a^{6}/ FRT2A, Sox21a^{6}
	w^{-}; Su(H)GBE-Gal4, UAS-nlsGFP, tub-Gal80^{TS}/UAS-upd2-RNAi^{NIG}; Sox21a^{6}/ FRT2A, Sox21a^{6}
	w^{-}; Su(H)GBE-Gal4, UAS-nlsGFP, tub-Gal80^{TS}/UAS-upd1-RNAi; Sox21a^{6}/ FRT2A, Sox21a^{6}
	w^{-}; Su(H)GBE-Gal4, UAS-nlsGFP, tub-Gal80^{TS}/UAS-upd3-RNAi; Sox21a^{6}/ FRT2A, Sox21a^{6}
	w^{-}; Su(H)GBE-Gal4, UAS-nlsGFP, tub-Gal80^{TS}/UAS-wg-RNAi^{GD}; Sox21a^{6}/ FRT2A, Sox21a^{6}
	w^{-}; Su(H)GBE-Gal4, UAS-nlsGFP, tub-Gal80^{TS}/UAS-wg-RNAi^{KK}; Sox21a^{6}/ FRT2A, Sox21a^{6}
	w^{-}; Su(H)GBE-Gal4, UAS-nlsGFP, tub-Gal80^{TS}/UAS-spi-RNAi; Sox21a^{6}/ FRT2A,

	Sox21a^{6}
**b**	w^{-}; MyolA-Gal4, UAS-nlsGFP, tub-Gal80^{TS}/+; Sox21a^{6}/ FRT2A, Sox21a^{6}
	w^{-}; MyolA-Gal4, UAS-nlsGFP, tub-Gal80^{TS}/UAS-upd3-RNAi; Sox21a^{6}/ FRT2A, Sox21a^{6}
	w^{-}; MyolA-Gal4, UAS-nlsGFP, tub-Gal80^{TS}/UAS-krn-RNAi; Sox21a^{6}/ FRT2A, Sox21a^{6}
**c**	w^{-}; +/+; FRT2A, Sox21a^{6}
**Supplementary Figure 9**	
**a**	w^{-}; btl-Gal4, UAS-actGFP/+; FRT2A, Sox21a^{6}/+
**b-f**	w^{-}; btl-Gal4, UAS-actGFP/+; FRT2A, Sox21a^{6}
**Supplementary Figure 10**	
**a**	w^{-}; Act5C-Gal4/UAS-GFP.SKL; Sox21a^{6}, tub-Gal80^{TS}/ FRT2A, Sox21a^{6}
**b**	w^{-}; EYFP-mito, Sox21a^{6}
**c,e**	w^{-}; +/+; FRT2A, Sox21a^{6}
**d**	w^{-}; gstD-lacZ/+; FRT2A, Sox21a^{6}
